# Aquatic Bacterial Communities Associated With Land Use and Environmental Factors in Agricultural Landscapes Using a Metabarcoding Approach

**DOI:** 10.3389/fmicb.2018.02301

**Published:** 2018-10-30

**Authors:** Wen Chen, Graham Wilkes, Izhar U. H. Khan, Katarina D. M. Pintar, Janis L. Thomas, C. André Lévesque, Julie T. Chapados, Edward Topp, David R. Lapen

**Affiliations:** ^1^Ottawa Research and Development Center, Science and Technology Branch, Agriculture and Agri-Food Canada, Ottawa, ON, Canada; ^2^Canadian Forest Service, Natural Resources Canada, Ottawa, ON, Canada; ^3^Ontario Ministry of the Environment and Climate Change, Environmental Monitoring and Reporting Branch, Toronto, ON, Canada; ^4^London Research and Development Centre, Science and Technology Branch, Agriculture and Agri-Food Canada, London, ON, Canada

**Keywords:** agricultural watersheds, aquatic bacterial community, land use, metabarcoding, stream order

## Abstract

This study applied a 16S rRNA gene metabarcoding approach to characterize bacterial community compositional and functional attributes for surface water samples collected within, primarily, agriculturally dominated watersheds in Ontario and Québec, Canada. Compositional heterogeneity was best explained by stream order, season, and watercourse discharge. Generally, community diversity was higher at agriculturally dominated lower order streams, compared to larger stream order systems such as small to large rivers. However, during times of lower relative water flow and cumulative 2-day rainfall, modestly higher relative diversity was found in the larger watercourses. Bacterial community assemblages were more sensitive to environmental/land use changes in the smaller watercourses, relative to small-to-large river systems, where the proximity of the sampled water column to bacteria reservoirs in the sediments and adjacent terrestrial environment was greater. Stream discharge was the environmental variable most significantly correlated (all positive) with bacterial functional groups, such as C/N cycling and plant pathogens. Comparison of the community structural similarity via network analyses helped to discriminate sources of bacteria in freshwater derived from, for example, wastewater treatment plant effluent and intensity and type of agricultural land uses (e.g., intensive swine production vs. dairy dominated cash/livestock cropping systems). When using metabarcoding approaches, bacterial community composition and coexisting pattern rather than individual taxonomic lineages, were better indicators of environmental/land use conditions (e.g., upstream land use) and bacterial sources in watershed settings. Overall, monitoring changes and differences in aquatic microbial communities at regional and local watershed scales has promise for enhancing environmental footprinting and for better understanding nutrient cycling and ecological function of aquatic systems impacted by a multitude of stressors and land uses.

## Introduction

Bacterial communities in freshwater are phylogenetically and metabolically diverse, being comprised of a complex mixture of common lineages within *Actinobacteria, Proteobacteria, Bacteroidetes, Verrucomicrobia, Planctomycetes*, and *Firmicutes*, candidate divisions (e.g., OD1, OP11, TM6, WS1, WS6), and genetic lineages unique to specific hydrological conditions (Zwart et al., 2002; Warnecke et al., 2004; Briée et al., 2007; Hu et al., 2014). Since the metabolism of bacterial communities contribute significantly to biogeochemical cycles (Newton et al., 2011; Staley et al., 2014; Amado and Roland, 2017), the capacity of an aquatic ecosystem to assimilate, dissipate, and ultimately be biologically resilient to pollutant pressures and hydro-physical changes, will depend largely upon the diversity and composition of the microbiota and their functional characteristics (i.e., photosynthesis and the oxidation, degradation, and/or fermentation of inorganic and organic substances; Das and Chandran, 2010; Schenker, 2014; Kumar, 2015). DNA-based work has been carried out to, assess the aptitude and effectiveness of both autochthonous and allochthonous microbes to degrade or utilize carbon resources (Das and Chandran, 2010; Jurelevicius et al., 2013; García-Armisen et al., 2014), detect sources of fecal pollution in water, and to evaluate the nature and status of other water quality properties and conditions (Ibekwe et al., 2013; Pandey et al., 2014; Ramírez-Castillo et al., 2015; Sales-Ortells et al., 2015; Sun et al., 2017).

Studies have shown that the diversity and structure of the aquatic microbiota can be shaped by stochastic dispersal and recruitment (i.e., mass effects) and environmental/species sorting (Brendan Logue and Lindström, 2008; Battin et al., 2016; Niño-García et al., 2016). Hydrological drivers (e.g., hydro-physiography, rainfall, and water flow; Llirós et al., 2014) and point or non-point-sources of bacteria (e.g., naturalized communities, land application of animal manure, or sewage discharge), shape aquatic bacterial community structure by influencing species introduction, dispersal, and residence time (Crump and Hobbie, 2005; Widder et al., 2014; Niño-García et al., 2016), and to a considerable extent, by modulating physiochemical properties of water like temperature and chemical status (Widder et al., 2014; Niño-García et al., 2016).

Localized sorting of aquatic bacterial communities in response to changes in water physiochemical properties has been previously reported (Moss, 2008; Hu et al., 2014; Sangchan et al., 2014; Staley et al., 2015; Van Rossum et al., 2015; Borges et al., 2017; Hosen et al., 2017). Jurelevicius et al. (2013) demonstrated that different *Proteobacteria* (e.g., *Achromobacter* spp.) and *Bacteroidetes* (e.g., *Cloacibacterium* spp.) taxa in freshwater were enriched in microcosms contaminated by certain petroleum derivatives. Similarly, contamination by the herbicide glyphosate stimulated the proliferation and growth of cyanobacteria (e.g., *Planktothrix* spp.; Saxton et al., 2011). The accumulation of autotrophic microbes can therefore be an indicator of excess nutrients (eutrophication; Miller et al., 2017). Recently, the abundance of *Polynucleobacter* and *Candidatus* Planktophila limnetica were found to be associated with elevated nitrogen levels in urban streams, while *Albidiferax*, a *Proteobacteria* genus was considered an indicator of tetrachlorethene contamination of groundwater (Hosen et al., 2017). These pollutants may act as nutrient sources or toxins, depending on bacterial functional lineages pre-existing at or near a water body (Saxton et al., 2011; Jurelevicius et al., 2013). Functional bacterial groups, such as denitrifiers, are largely affected by the degree of nutrients, oxygen, temperature, pH, and alkalinity of water (Lindström et al., 2005; Hu et al., 2014; Llirós et al., 2014; Huang et al., 2015; Staley et al., 2015; Le et al., 2016). Some of these studies have associated aquatic microbial community compositional structure with hydrology and physiochemical properties of water (Staley et al., 2013, 2014, 2015; Niño-García et al., 2016; McCarthy et al., 2017). However, due to the large number of usually dynamic and interacting factors that can affect community structure in natural systems, it is difficult to identify precisely causal effects (Lear and Lewis, 2009; Wang et al., 2011; Garrido et al., 2014; Van Rossum et al., 2015).

By using metabarcodes of bacterial 16S rRNA genes, we aimed in this paper to (1) characterize the taxonomic and functional profiles of microbial communities of surface waters from mixed-use but primarily agriculturally-dominated watersheds in eastern Canada; (2) evaluate potential associations among bacterial taxa and functions with a suite of environmental and land use factors; (3) frame these associations in the context of the surface water systems' capacity to cope with future and concurrent stressors; and (4) determine if microbiota compositional structures based on 16S rDNA metabarcodes can serve as bioindicators of stream health and/or condition in agriculturally-dominated freshwater systems.

## Materials and methods

### Study site description, water sampling, and analyses

**Figure 1:**
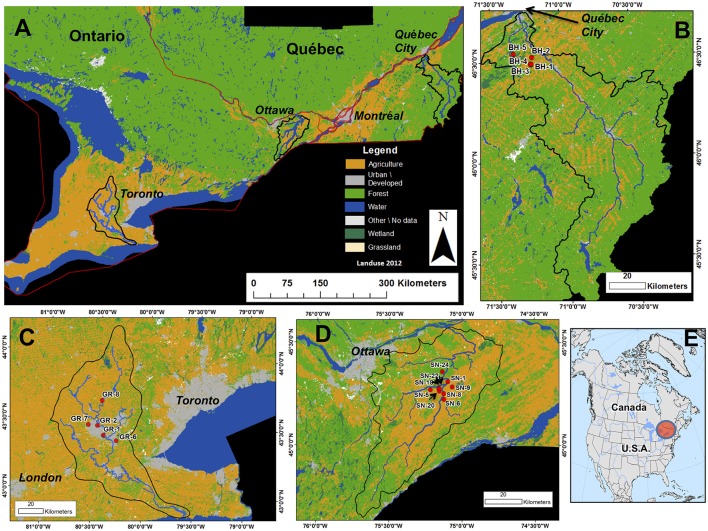
The sampling sites in the current study. **(A)** Southern Ontario and Québec; **(B–D)** the sampling sites at **(B)** the Bras d'Henri (BH) River, **(C)** the Grand River (GR); and **(D)** the South Nation River area (SNRA); **(E)** the relative location of the sampling sites in North America.

The environmental and land use variables associated with each water sample are described in Table 1 and the geographic locations of the sampling sites are shown in Figure 1.

**Table 1:** Land use and water physiochemcial/environmental variables used for data mining.

**Variable name**	**Variable description (unit)**
SITE_ID	Independent variable site identifier
STRAHLER	Numeric size of sample site stream/watercourse (Strahler, 1952). Larger values indicate larger water courses.
SEASON	Sampling time based on solstice and equinox dates
WAT_AMIA_AMN	NH_3_ (ammonia) + NH_4_ (ammonium) concentration in sample water (mg L^−1^)
WAT_SUSSOL	Suspended sediments/solids in sample water (mg L^−1^)
WAT_NITRATE	NO${}_{3}^{-}$ (nitrate) concentration in sample water (mg L^−1^)
WAT_REA_PHOS	Reactive phosphorus concentration in sample water (mg L^−1^)
WAT_TOTKN	Total Kjeldahl nitrogen (TKN) concentration in sample water (mg L^−1^)
WAT_TOTPHO	Total phosphorus concentration in sample water (mg L^−1^)
WAT_TEMP_C	Temperature of sample water (°C)
WAT_PH	pH of sample water
WAT_CONDUCTIVITY_MSC	Specific conductivity of sample water (mS cm^−1^)
WAT_DISS_OXYGEN_MGL	Dissolved oxygen in sample water (mg L^−1^)
WAT_ORP_MV	Oxidation reduction potential of sample water (mV)
WAT_TURBIDITY_NTU	Cloudiness of sample water as measured with a nephelometer sensor (NTU; nephelometric turbidity units)
RU_DISM3S	Mean daily river discharge at Russell hydrometric station (m^3^ s^−1^)
WEBS_RAIN_MM WEBS_RAIN_MM_xD	Total rainfall at WEBs for day of sampling; total rainfall for day of sampling and × = 1, 2, 3, and 5 days in advance of sampling day (mm)
WEBS_MX_TEMP_CWEBS_MIN_TEMP_C	Daily maximum, minimum air temperature at WEBs
WEBS_SLR_RAD_WM2	Daily average incoming solar radiation at WEBs (W m^−2^)
BASIN_(land use)	Proportion of year 2012 land use (determined via RS) in total sample site catchment area: agriculture (BASIN_AGRICULTURE), urban/developed (BASIN_URBDEV), treed/forest (BASIN_TREE), wetland (BASIN_WETLAND), and other land uses (BASIN_OTHER) (km^2^ km^−2^)
DEVELP_05K	Proportion of developed land, identified via RS, in catchment areas upstream of site with MUFL of 5 km (km^2^ km^−2^) in year in which sample was collected
AG_05K	Proportion of agricultural land, identified via RS, in catchment areas upstream of site with MUFL of 5 km (km^2^ km^−2^) in year in which sample was collected

*All variables (sans SITE_ID) were used as independent predictor variables in CART analyses. x, number of days in advance of sample; K, km; RS, remote sensing; MUFL, maximum upstream flow length*.

#### South nation river area (SNRA)

Nine water sampling sites were selected based on divergent contributing land uses, stream size, and function (Figure 1d, Table 2). The study sites have been described previously (Ruecker et al., 2007; Wilkes et al., 2011, 2013b; Lapen et al., 2016; Jones et al., 2017). Briefly, the ~3,900 km^2^ South Nation River basin is located in Eastern Ontario, Canada (Figure 1d). Nearly 60% of the SNRA basin was classified as agricultural land in 2012. Dairy livestock farming is common in this region, where applications of solid and liquid livestock manure on fields in the spring and fall are routinely conducted.

**Table 2:** Bacterial diversity indices at each watershed.

										**Diversity index (MEAN** ±**SE)**				
													**True-diversity**	
**SITE_ID**	**Description**	**Upstream contributing Area (km^2^)**	**STRAHLER Stream order**	**Agriculture**	**Urban/Developed**	**Forest**	**Wetland**	**Other**	**Number of samples**	**Coverage**	**OTU number**	**Chao1**	**Shannon-Wiener**	**Gini-Simpson**
				**(% land)**	**(% land)**	**(% land)**	**(% land)**	**(% land)**						
**SOUTH NATION RIVER AREA**														
SN_1	Drinking water intake (~6 m from surface)	2371	9	52	2	42	3	2	10	0.99 ± 0	276 ± 23	560 ± 69	30 ± 2	14 ± 1
SN_5	Tributary, Mixed Urban/Agricultrue development	81	6	65	2	30	1	2	15	0.97 ± 0.01	328 ± 72	968 ± 251	36 ± 19	13 ± 5
SN_6	Tributary, Mixed Urban/Agricultrue development	176	7	54	1	43	1	1	12	0.97 ± 0.02	262 ± 57	737 ± 267	23 ± 9	8 ± 1
SN_8	Main river^*^	1413	8	49	1	46	3	1	3	0.99 ± 0	235 ± 29	382 ± 30	32 ± 6	15 ± 3
SN_9	Tributary, Mixed Urban/Agricultrue development	54	6	72	1	23	4	1	1	0.99±*NA*	96±*NA*	190±*NA*	9±*NA*	5±*NA*
SN_18	Agriculture stream (Dairy cattle farms)	<5	3	90	0	10	0	0	11	0.92 ± 0.03	504 ± 130	1491 ± 327	110 ± 79	41 ± 33
SN_20	Agriculture stream	<5	4	90	0	9	0	1	8	0.89 ± 0.04	500 ± 120	1383 ± 304	82 ± 35	26 ± 13
SN_22	Agriculture stream (Dairy cattle farms)	<5	3	90	0	10	0	0	1	0.89±*NA*	256±*NA*	664±*NA*	35±*NA*	10±*NA*
SN_24	Forest/wetland^*^ (wildlife)	<5	4	0	0	100	0	0	10	0.97 ± 0.01	378 ± 94	1059 ± 244	24 ± 10	7 ± 2
**BRAS D'HENRI (BH)**														
BH_1	Tributary of BH-5	2.4	2	85	0	14	0	1	1	0.81±*NA*	544±*NA*	1520±*NA*	188±*NA*	64±*NA*
BH_2	Tributary of BH-5	4.2	2	80	0	20	0	2	1	0.62±*NA*	736±*NA*	2690±*NA*	333±*NA*	94±*NA*
BH_3	Agriculture stream of BH-1	1.6	1	78	0	21	0	1	2	0.87 ± 0.1	493 ± 276	1426 ± 991	97 ± 67	19 ± 5
BH_4	Agriculture stream of BH-1	0.8	1	99	0	0	0	1	2	0.74 ± 0.01	1050 ± 430	2941 ± 1226	448 ± 170	133 ± 20
BH_5	Main river	167	3	66	5	25	2	2	1	0.83±*NA*	779±*NA*	1562±*NA*	311±*NA*	99±*NA*
**GRAND RIVER (GR)**														
GR_1	Drinking water intake	2485	7	74	6	15	3	2	4	0.99 ± 0	226 ± 28	408 ± 64	26 ± 2	12 ± 1
GR_2	WWTP outflow	–		–	–	–	–	–	3	0.9 ± 0.01	224 ± 39	733 ± 150	31 ± 3	12 ± 3
GR_6	Recreation beach (Shade Mill)	100	6	34	7	50	2	8	3	0.99 ± 0	176 ± 27	314 ± 71	28 ± 4	14 ± 3
GR_7	Recreation beach (Laurel Creek)	31	5	48	14	34	1	3	3	0.99 ± 0	211 ± 28	384 ± 48	21 ± 2	9 ± 1
GR_8	Recreation main river (Elora Gorge)	1020	7	71	2	17	7	2	3	0.95 ± 0.03	238 ± 36	652 ± 65	31 ± 8	12 ± 4

* *Land use summaries for year 2013*.

The nine SNRA sampling sites are located in surface water catchments ranging from <5 to ~2,400 km^2^ (Table 2). Sites SN_18, 20, and 22 are located on smaller watercourses (ditches) fed primarily by agricultural sub-surface tile drainage (Sunohara et al., 2016). SN_5, 6, and 9 are intermediate tributaries feeding the main river stem directly, and are impacted by mixed agricultural activities and some urbanization. SN_1 and 8 are located on the main stem of the South Nation River. SN_1 is a drinking water intake for a small community, and the water is extracted at ~6 m depth below the river surface. Site SN_24 is located on a small stream that drains from a forested area not impacted by any known anthropogenic land use activity. All SNRA water samples were collected bi-weekly starting in spring and ending in fall for 3 years (2010–2012), representing a total of *n* = 73 water samples. There was only one sample from SN_22 and SN_9, and only three samples from SN_8. The other sites had >8 samples collected.

All water samples were collected in 2 L sterile Polyethylene Terephthalate (PET) bottles (Systems Plus, Baden, Ontario) and returned on ice to Agriculture and Agri-Food Canada's (AAFC) Ottawa Research and Development Centre (ORDC, Ottawa, Canada) laboratory and processed for microbiological analysis within 24 h of collection. Water temperature, pH, dissolved oxygen, specific conductivity, and turbidity were determined using a YSI 6600 multi-parameter water quality sonde (YSI Inc., Yellow Springs, OH) following manufacture's instruction(Wilkes et al., 2011). Water samples were also split and sent to the Robert O. Pickard Environmental Centre (ROPEC, Ottawa Canada) laboratory for nitrogen, total phosphorus, and suspended solids analysis as described previously (Wilkes et al., 2011).

#### Bras d'henri (BH)

There were a total of five sampling sites from the Bras d'Henri (BH) River drainage area near Quebec City, Quebec, Canada: sites BH_1, 2, 3, 4, and 5. These sites are impacted variably by mixed farming and intensive swine production (Figure 1b). Agricultural land area exceeds ~66% for these sampling basins (see Table 2). Upstream land uses for sites BH_1–3 and 5 are ~14–25% tree/forest. The upstream catchment area associated with BH_4 is almost entirely agricultural land under livestock cropping systems. BH_5 is located on the outlet of the BH River (Figure 1b). Drainage areas of sites BH_1–4 are <5 km^2^ while BH_5 has a drainage contributing area of ~170 km^2^ (AAFC, 2018). A total of seven (*n* = 7) samples were collected at the BH sites during the fall of 2012.

#### Grand river (GR)

The extent of the Grand River (GR) basin and the five water sampling sites utilized in this study are given in Figure 1c. The sites are composed of three water recreational sites, including Laurel Creek (GR_7) and Shade's Mills (GR_6) which are located on tributaries of the GR, and the Elora Gorge (GR_8) site located on the main stem of the GR. Two additional sites on the GR were sampled, one upstream of a drinking water intake (GR_1) and one downstream of the outflow from a wastewater treatment plant (WWTP) (GR_2). A total of 16 surface water samples were collected between June and September in 2011. Land uses upstream of GR_1 and GR-8 exceed 70% agricultural land; whereas GR_6 and GR_7 are associated with a mix of both agricultural (34–48%), forest (34–50%), and urban (7–14%) upstream land uses (Table 2).

All BH and GR water samples were collected in 1 L sterile PET bottles (Systems Plus, Baden, Ontario) and express shipped on ice to AAFC's ORDC laboratory and processed for microbiological analysis within 24 h of collection.

### Land use, stream order, and hydrological/meteorological variables at SNRA

Land use variables (Table 1) were generated following the methods previously described by Wilkes et al. (2011). SNRA had the most water samples and the densest ancillary data for multivariate analyses, and as such, the subsequent discussion of methods and approaches in this section apply only to SNRA.

Land use data in the form of raster layers was obtained from AAFC's Annual Crop Inventory for each year of this study (AAFC, 2017). Surface water catchment areas (entire catchment and catchment 5 km from the sample site) and flow direction were determined within ArcMap 9.2 (Environmental Systems Research Institute, Redlands, CA). Land use was classed as agricultural land, urban/developed land, treed land, and wetland as well as an “other” category. Total upstream catchment areas were summarized for all study basins for year 2012 (Table 2). Stream order (Strahler, 1952) was determined via methods already discussed previously (Lyautey et al., 2010, 2011).

Samples were assigned a seasonal category relative to solstice and equinox dates (variable: SEASON). Daily maximum and minimum temperature, solar radiation, and total daily rainfall were monitored at a HOBO (Onset Computer Corp., Bourne MA) instrumented weather station near SN_20 (Sunohara et al., 2015); also known as location. Cumulative rainfall estimates were calculated on the day of sampling and 1, 2, 3, and 5 days prior to sampling (Lyautey et al., 2010; Wilkes et al., 2011). Mean daily discharge (m^3^ s^−1^) was acquired from the Water Survey of Canada (2017) (WSC, 2017) for Russell Ontario's gauging station as a proxy for all sites.

### DNA extraction, PCR amplification, and 454 pyrosequencing

DNA extraction, PCR amplification, and 454 pyrosequencing library preparations were carried-out at the core sequencing facility at ORDC, AAFC (Ottawa, Canada) laboratories. For DNA extraction, 250–500 mL (depending on the degree of turbidity) of water was filtered through 0.22 μm membrane filters and total DNA was extracted using PowerSoil® DNA extraction kits following manufacturer's instructions (MoBio Laboratories, Inc., CA). The concentration and quality of the extracted genomic DNA were assessed using a NanoDrop ND-1000 Spectrophotometer (Nanodrop Technologies, Inc., DE). Samples with low DNA yield were further concentrated using the Savant DNA 120 SpeedVac Concentrator (Thermo Fisher Scientific, CO).

Genomic DNA extracted from environmental samples was serially diluted in order to reduce the effects of PCR inhibitors. The bacterial 16S rDNA was amplified by a universal primer pair, UN-BacF (Forward: 5′-GAT CCT GGC TCA GGA TGA AC-3′) and UN-BacR (Reverse: 5′-GGA CTA CCA GGG TAT CTA ATC-3′), targeting the hypervariable V1–V4 regions (*ca*. 900 bp) (Xu et al., 2010). The forward and reverse primers were fused with unique 24-nucleotide MID (Roche Multiplex Identifiers) barcodes to avoid individual adaptor ligations for each library (Supplementary Table S1). Eight replicates of low-cycle PCR amplicon libraries were pooled together to limit the effects of PCR bias and to increase the detectable biodiversity in each sample. Amplicons were purified using a PureLink PCR Micro Purification Kit (Life Technologies Inc., Grand Island, NY) and eluted in 18 μL of pre-warmed (60°C) elution buffer. The absence of primer dimers was confirmed by Bioanalyzer electropherograms (Agilent Technologies Inc., CA). Each purified amplicon library was quantified using a Nanodrop ND-1000 Spectrophotometer (Thermo Fisher Scientific Inc., MA), normalized to 50 ng μL^−1^ and used for sequencing reactions. For each pool, 24 equimolar MID-tagged amplicon libraries were combined. All amplicons in each of the pools were then ligated with 454-Pyrosequencing adaptor L and were sent to National Research Council-Plant Biotechnology Institute (NRC-PBI, Saskatoon, Canada) for unidirectional pyrosequencing.

### High throughput sequencing data processing

The raw pyrosequencing data were demultiplexed using unique barcodes (MID; Supplementary Table S1) and denoised using the shhh.flow function in Mothur (version 1.30.2; Schloss et al., 2009; Quince et al., 2011). Sequences were trimmed or removed when exhibiting ambiguous letters, an average quality scores lower than 25 per 50 bp, length shorter than 325 or longer than 1,200 bp, and/or maximum homopolymers longer than 7. Operational Taxonomic Units (OTUs) were picked for all remaining sequences using QIIME pick_closed_reference_otus.py function against the greengenes 16S rDNA database (DeSantis et al., 2006) pre-clustered at 97% similarity (version gg_13_5), representing putative bacterial species (Stackebrandt and Goebel, 1994; Schloss and Handelsman, 2006; Koeppel and Wu, 2013). Singleton OTUs (OTUs containing only one sequence) were removed from the dataset to reduce diversity inflation (Huse et al., 2010). The remaining OTUs were normalized by their predicted 16S rRNA gene copy number, thus transfering the 16S rRNA operon abundance to species abundance (Kembel et al., 2012; Langille et al., 2013). The abundance of each normalized OTU was round up to the next integer to preserve recovered taxonomic information. The OTUs were further assigned to functional groups using the FAPROTAX database (Louca et al., 2016).

To infer the natural habitats of microbes recovered in the current study, metabarcodes were BLASTed (Camacho et al., 2009) against MetaMetaDB (Yang and Iwasaki, 2014), a database containing close to three million prokaryotic 16S rDNA sequences pre-categorized to 61 environmental categories (including nested groups) based on public metagenomics datasets.

The 454 pyrosequencing sequences from all 96 surface water samples are available through the Sequence Read Archive (SRA) under study accession number PRJNA322351 with sample accession numbers SAMN05159069 to SAMN05159164.

### Statistical analysis

Most statistical analyses were completed with R (R Core Team, 2017). Diversity indices and community compositional structure were calculated and described for all samples (from SNRA, BH, and GR). Only SNRA samples, supported by a wide range of ancillary data, were subjected to multivariate analyses.

Discrimination of SNRA sampling sites on the basis of ancillary independent data was assessed by partial least squares discriminant analysis (PLS-DA**;** Kurtz, 2014). To calculate the alpha-diversity indices, functions in vegan (Oksanen et al., 2018), BiodiversityR (Kindt and Coe, 2005), and iNEXT (Hsieh et al., 2016) were used. We converted Shannon entropy (Shannon-Weiner, SW) and Gini-Simpson (GS) indices to true diversities (TD) (Jost, 2006), represented by acronyms SW-TD and GS-TD, respectively, using formulas: SW-TD = exp(SW); GS-TD = 1/(1-GS). In comparison with regular alpha-diversity indices, TD allows for direct comparison and interpretation of real diversity (Jost, 2006).

The OTU abundance matrix was hellinger-transformed for subsequent analysis (Legendre and Gallagher, 2001). To assess the multivariate homogeneity of group variances, “betadisper” followed by “permutest” using default settings was performed. To quantify variances in community composition between groups of sampling units, analysis of similarities (ANOSIM) was used with permutations being constrained within sampling year or season. The redundancy analysis (RDA) was performed using function cca in vegan. Kruskal-Wallis rank sum tests were performed to assess the differences in abundance of OTUs, taxa at different ranks, and functional groups in different environmental niches, using “kruskal.test” in R. The Kruskal-Wallis results were visualized in heat-maps using pheatmap function (Kolde, 2015). Correlations between the relative abundance of functional groups and environmental attributes were assessed with Spearman's rank correlations (Spearman's rho). The influence of environmental variables on the divergence of compositional structure of each functional group was assessed by partial RDA using cca in vegan. The proportion of community compositional variance explained by each factor was represented by an adjusted *R*^2^.

To assess if bacterial community structure can be used as a bioindicator of water quality, a network was constructed based on the distance matrix using the hellinger-transformed OTU abundance table and was visualized using R package qgraph (Epskamp et al., 2012). The ecological association (correlation-based relevant) networks of OTUs were constructed using the random matrix theory (RMT)-based approach implemented in the Molecular Ecological Network Analysis (MENA) pipeline available at http://ieg4.rccc.ou.edu/mena (Deng et al., 2012). Different from other network construction methods where correlation coefficient thresholds are arbitrarily selected based on empirical knowledge, RMT-based approaches reveal coexisting patterns of community members by successively removing small values of correlation coefficients (weaker correlations) and identifying the critical transition point where the distribution of eigenvalue spacing of correlation matrices changes from Wigner-Dyson to Poisson statistics (Luo et al., 2006; Zhou et al., 2010, 2011; Deng et al., 2012), and therefore is more objective (Schmitt et al., 2004; Deng et al., 2012). The 97%-identity OTUs with more than 30 reads and recovered from 50% of samples in a given dataset were hellinger-transformed and subjected to correlation analyses using the Spearman rank approach. The network was constructed using correlation thresholds determined by RMT and by the fast greedy modularity optimization method. The topological properties of the empirical molecular ecological network (MEN) and the corresponding random MENs with identical network sizes and average number of links were calculated and compared. Eigengene analysis was performed to evaluate the impact of environmental traits in determining module profiles and network interactions. Networks were visualized in cytoscape (v. 3.6.1) (Shannon et al., 2003; Cline et al., 2007; Lotia et al., 2013) and Gephi (v. 9.3) (Bastian et al., 2009).

R packages RAM (Chen et al., 2016), ggplot2 (Wickham, 2011), phyloseq (McMurdie and Holmes, 2013), lattice (Sarkar, 2008), corrplot (Wei, 2013), grid (Murrell, 2013) gridExtra, ggord (Beck, 2016), and ggbiplot (Vu, 2011) were used for transforming and manipulating OTU tables and visualizing statistical results.

The relationships between SW-TD (true diversity converted from Shannon entropy) of aquatic microbiota and the suite of independent environmental variables were examined by Classification and Regression Tree Analyses (CART) (CART® Pro 6.0 Salford Systems, San Diego, CA). CART was conducted using the least square (LS) regression tree mode. CART analyses and approaches used herein have been described in detail previously (Wilkes et al., 2009, 2011, 2013b).

## Results

In total, 1,520,569 high-quality reads (gene abundance) were clustered to 5,423 greengenes reference OTUs at 97% sequence identity cut-off. The organismal abundance (867,607), obtained by dividing the gene abundance of each OTU by its predicted 16S rRNA gene copy number (Kembel et al., 2012; Langille et al., 2013) was used to summarize the diversity of bacterial communities (Table 2). The sample coverage (completeness) indices (Table 2, all sampling sites) and the species accumulation curves (Supplementary Figure S2C, SNRA data only) suggest a near-saturation of species recovery at each sampling site.

### Bacterial community at SNRA watersheds

A clear, quantitative discrimination of sample sites based on independent criteria listed in Table 1 is demonstrated by sPLS-DA analysis (Supplementary Figures S1A,B). Agricultural sites SN_18 and 20 were grouped together, which are geographically close and were associated with higher turbidity, rainfall, and nutrients. Sites SN_5, 6, and 8 were also grouped, showing stronger association with mixed agriculture and urban development. SN_1 was associated with higher water temperature and urban development, while reference site SN_24 was associated with higher tree coverage.

#### Association between bacterial community diversity and environmental attributes

Overall, the observed diversity, and its standard deviation and error, were higher at agriculturally dominated drainage ditch sites (SN_18, 20, and 22) with lower stream order, compared to larger stream order sites downstream of more diversified land uses (Table 2, Supplementary Figure S2C). More OTUs were shared by samples from higher (STRAHLER = 8–9, 114 OTUs) and medium (STRAHLER = 5–7, 64 OTUs) order systems, relative to lower (STRAHLER = 3–4, 22 OTUs) order streams (Supplementary Figures S2A,B). The Shannon-Weiner-based true diversities (SW-TD) of SNRA samples ranged from 5 to 110, compared to 19–448 and 9–31 for BH and GR samples, respectively (Table 2).

**Figure 2:**
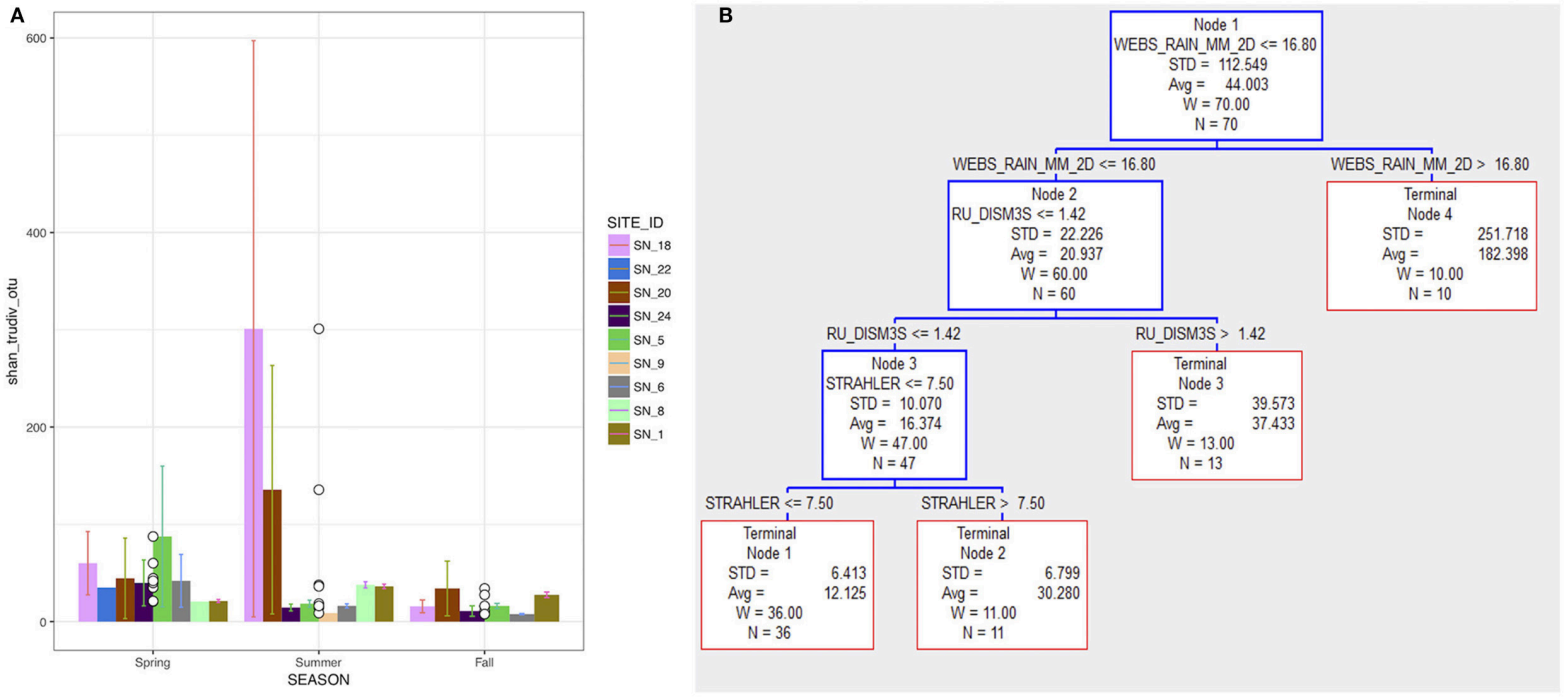
**(A)** The true diversity converted from Shannon-Wiener index (TD-SW) of bacterial communities at each SNRA site. **(B)** CART regression tree predicting TD-SW from independent variables given in Table 1.

Excluding SNRA sites with ≤ 3 samples, the highest diversity was found at site SN_18, a ditch that receives drainage waters from agricultural cropping systems. The lowest diversity was found within medium order tributaries with more diversified, but predominately agricultural upstream land uses (SN_5&6), and site SN_24 where there was no known anthropogenic land use upstream. Seasonality of the SW-TD is given in Figure 2A. At the agriculturally dominated sites SN_18 and 20, higher diversity was observed in the summer, which was influenced in large part by diversity of samples collected in late August following a large rainfall event. The SW-TD of these specific post-rain event samples at site SN_18 and 20, were 893 and 263, respectively. The average diversity of other samples collected at these two sites in summer was 5.

CART analysis of the SNRA data showed that SW-TD was optimally classified by hydrological driving variables and stream order (Figure 2B); relative to the other independent variables used as predictors (Table 1). Node 1 SW-TD data were split on the basis of 2-day cumulative rainfall (WEBS_RAIN_MM_2D) where samples collected during >16.8 mm (Terminal Node 4) were associated with a greater diversity (SW-TD avg. ± SD = 252 ± 182) in relation to those classed in Node 2 (21 ± 22) where WEBS_RAIN_MM_2D ≤ 16.8 mm. The cumulative 2-day rainfall variable was used as a predictor in CART to capture potential hydrological lag effects associated with the mobilization of bacteria to/in the stream/river courses. The split of SW-TD data in Node 2 indicates that under relatively lower WEBS_RAIN_MM_2D (≤ 16.8 mm) and relatively higher river discharge (RU_DISM3S) (>1.42 m^3^ s^−1^) SW-TD was relatively higher (Terminal Node 3). This is in relation to Node 3 SW-TD group of data where discharge was relatively lower (RU_DISM3S ≤ 1.42). And finally, CART split Node 3 data into two terminal nodal SW-TD groups on the basis of stream order, where average SW-TD was relatively lower while stream order was also relatively lower (STRAHLER ≤ 7.5). Nevertheless, without context dependent classification of the SW-TD in Figure 2B, the site comparison findings in Table 2 suggest, overall, that smaller tributaries had greater diversity.

#### Dynamics of the taxonomic compositional structure

**Figure 3:**
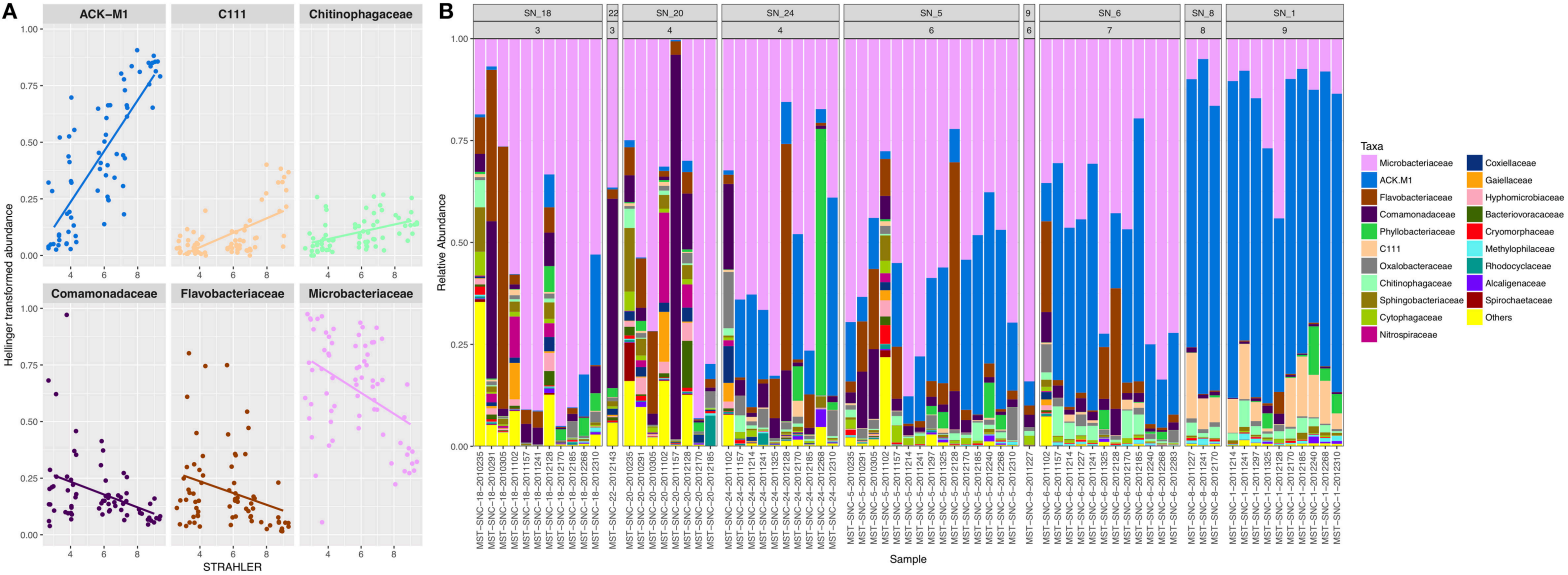
The taxonomic compositional structure of bacterial communities. **(A)** The correlations between STRAHLER stream order and the relative abundance of bacterial families. **(B)** The relative abundance of the top 20 most abundant bacterial families. The panel labels of the faceted plot represent sampling site IDs (first row) and STRAHLER stream order (second row). Only one sample was collected from each of the following two sites: SN_22 and SN_9.

At SNRA, the surface water was dominated by *Actinobacteria* (81.5% total abundance, 17.97% OTUs), which was recovered to a significantly greater degree at the large watercourses relative to the smaller ditch/tributaries (*p* < 0.001). All of the most prevalent OTUs were members of *Actinobacteria*, with three assigned to *Microbacteriaceae*: *Candidatus* Rhodoluna (0.0007–2%), *Clavibacter* (0–0.64%), and an unclassified sp. (0.0002–4%); and two were assigned to the ACK-M1 family (0–1.3 and 0–0.67%, respectively). Other recovered bacterial phyla were <10% in abundance, including *Proteobacteria* (7.8%), *Bacteroidetes* (7.6%), and some candidate divisions such as TM7 (1.7%) and OD1 (0.3%). Figure 3 shows that *Microbacteriaceae* and ACK_M1 were ubiquitously distributed but the abundance of the former decreased, while that of the latter increased, with stream order (*p* < 0.001). At a significance level of 0.01, *Deltaproteobacteria, Spartobacteria* (including *Chthoniobacterales* and *Chthoniobacteraceae*), *Planctomycetales* (including *Planctomycetaceae*), *Rhodospirillaceae* and *Legionellaceae* (including *Rickettsiella), Salinibacterium*, and *Hyphomicrobium* were more abundant in lower and medium order streams, while *Candidatus* Aquiluna was more frequently recovered in the higher order streams.

**Figure 4:**
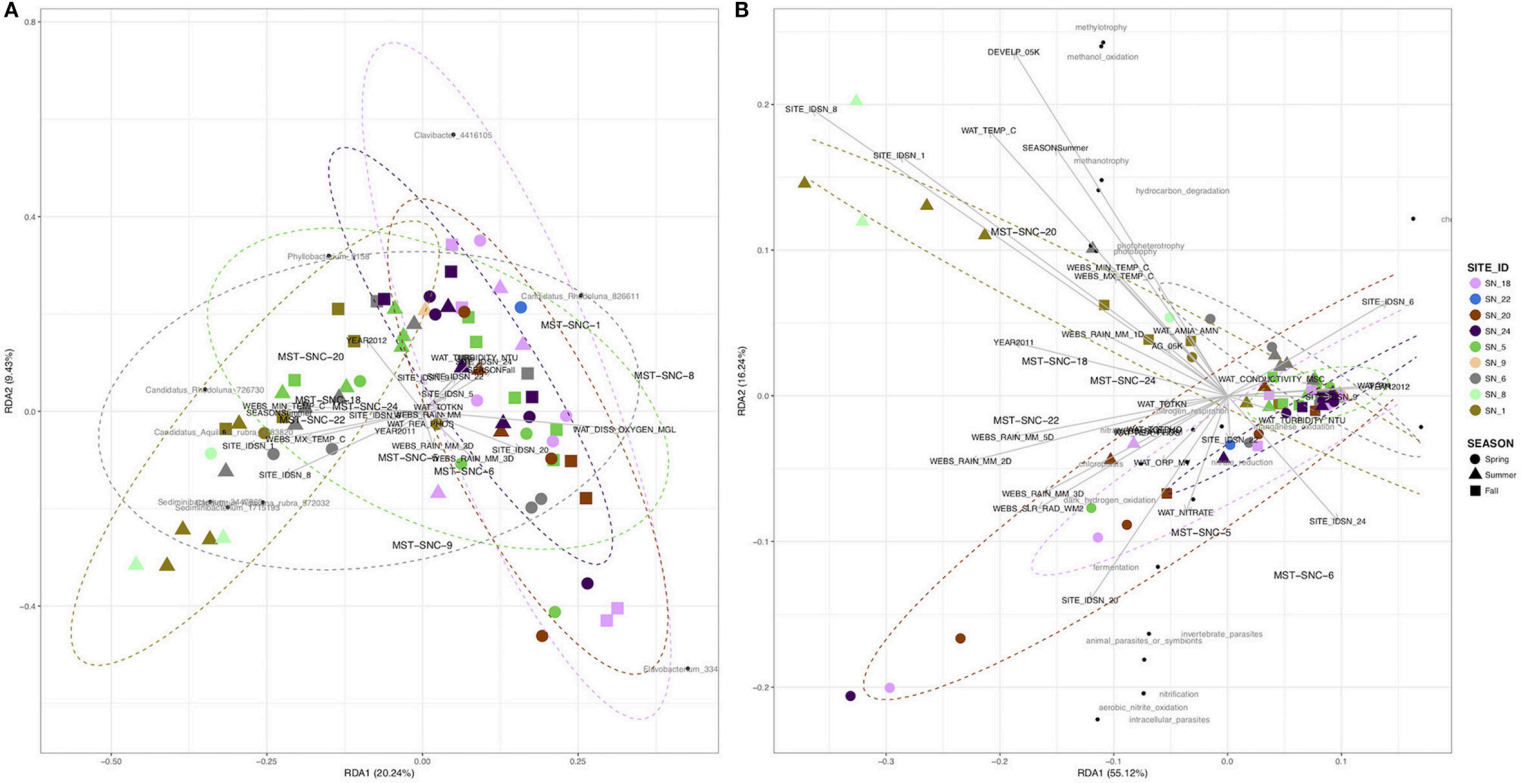
RDA of surface water bacterial community at SNRA based on **(A)** Hellinger-transformed OTUs, and **(B)** relative abundance of functional groups identified by FAPROTAX 1.0.

The RDA showed that the total explainable variance (EV) in community composition was 49.1%. The dominant explanatory variables were stream order (28.4%), season (6.6%), mean daily river discharge (RU_DISM3S) (3.8%), year (3.5%), daily minimum air temperature (WEBS_MIN_TEMP_C) (3.5%), proportion of agricultural land upstream of sampling site (AG_05K) (1.9%), and total Kjeldahl nitrogen (WAT_TOTKN) (1.7%). Figure 4A shows that water samples from small agricultural drainage ditches (SN_18, 20, and 22) were separated from those of main river stems (SN_1 and 8), while samples from medium order streams impacted by mixed land uses (SN_5, 6, and 9) were intermingled in the two dimensional space. The RDA also suggests that variance in bacterial community composition at the small agricultural ditches was associated with higher cumulative rainfall and dissolved oxygen in water, while in the main river stems, it was influenced more by water temperature. Permutation tests for homogeneity of multivariate dispersions (Supplementary Figure S3) suggest higher beta-diversity at lower order streams (*p* ≤ 0.001). Sampling year and season, however, showed insignificant or marginal effects on community structure based on ANOSIM (Supplementary Figures S4A,B).

By BLASTing against MetaMetaDB (Yang and Iwasaki, 2014) at 97% sequence identity, we further noticed that soil associated bacteria were more abundant in the smaller agricultural drainage ditches than at the main river stem sites, irrespective of the diversity of bacterial communities. For example, soil bacteria recovered from the water samples with the highest diversity at SN_18 (SW-TD = 893) and SN_20 (263) were more abundant than those from SN_1 (SW-TD = 43) and SN_8 (35) by approximately one order of magnitude (Supplementary Figure S5). Soil bacteria were also more abundant in samples with the lowest diversity at SN_18 and 20 (SW-TD = 3 and 2, respectively) than in samples with the highest diversity from SN_1 and 8 (SW-TD = 43 and 35, respectively).

#### Functional bacterial groups

**Figure 5:**
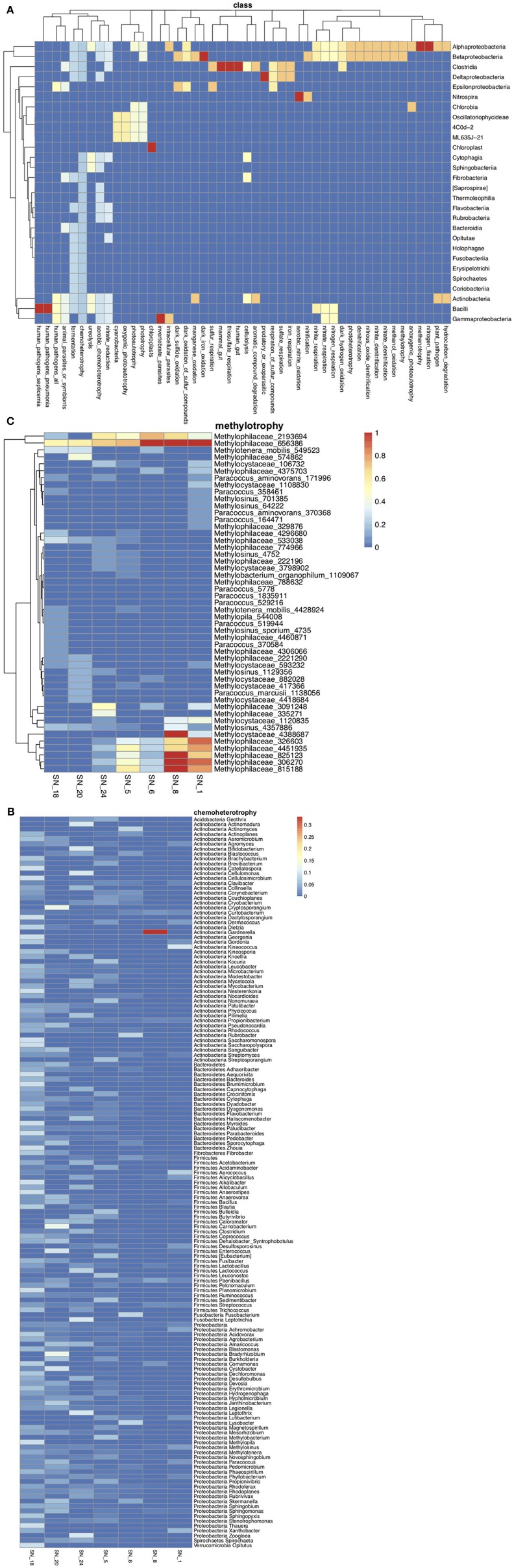
**(A)** OTU richness in each functional group at the class level. The warmer color represents higher OTU numbers from a class contributed to a function. **(B)** The average relative abundance of chemoheterotrophic genera at each sampling site. The genera are grouped by the phyla they belong to. **(C)** The average relative abundance of OTUs belonging to methylotrophic bacteria.

FAPROTAX annotated 1,293 out of 4,731 OTUs to 68 known functional groups. Although *Actinobacteria* was the most abundant group, members of *Alphaproteobacteria* and *Betaproteobacteria* contributed to a broader range of metabolism pathways or ecological functions (Figure 5A). The most abundant and diverse functional group was chemoheterotrophs, containing 944 OTUs representing 149 genera, among which, *Clavibacter* (56%), *Flavobacterium* (27%), and *Phyllobacterium* (9%) had the highest relative abundance and were found across all sampling sites (Figure 5B). It should be noted that many OTUs assigned to chemoheterotrophic bacteria were also potentially associated with other processes, such as fermentation, cellulolysis, xylanolysis, methanol oxidation, and the metabolism of nitrogen, sulfate and phosphorus. The chemoheterotrophic group had higher richness and beta-diversity at low to medium order streams (Figure 5B). This trend was also observed for bacteria involved with cellulolysis (e.g., *Cytophaga* spp. and *Dyadobacter* spp., Supplementary Figure S6A), nitrification (e.g., *Nitrospira* spp., Supplementary Figure S6B), respiration of sulfur (e.g., *Desulfovibrio* spp. and *Desulfobulbaceae* spp., Supplementary Figure S6C), fermentation (e.g., *Paludibacter* spp., Supplementary Figure S6D), and those categorized as animal parasites or symbionts (e.g., *Rickettsiella* spp. and *Staphylococcus* spp., Supplementary Figure S6E). On the other hand, sequences affiliated to the methylotrophic *Methylophilaceae* spp. (Figure 5C) were more diverse and abundant at main river stems (SN_1 & 8), while *Paracoccus* spp. were more abundant at lower order agricultural streams. Only a single *Methylophilaceae* OTU (Methylophilaceae_656386) was recovered from all SNRA sampling sites.

The RDA derived EV was much smaller for community functional structure (EV = 10%) than for taxonomic structure (EV = 49%). The EV was accounted for primarily by stream order (28%, *p* < 0.001), river discharge (RU_DISM3S) (13.9%, *p* < 0.001), dissolved oxygen in water (WAT_DISS_OXYGEN_MGL) (2.8%, *p* < 0.01), total rainfall for 5 days prior sampling (WEBS_RAIN_MM_5D) (2.5%, *p* < 0.05), and ammonia/ammonium concentration (WAT_AMIA_AMN) (2.1%, *p* < 0.05) (Figure 4B). In addition, the abundance of 49 functional groups was significantly (*p* ≤ 0.05) correlated to one or more environmental attributes (Table 1), but most of the correlations were weak to moderate [Spearman's rho ± standard error (SE): 0.36 ± 0.01]. The strongest positive correlation was between the stream order (STRAHLER) and the abundance of methylotrophic bacteria (Spearman's rho = 0.61, *p* < 0.001). River discharge (RU_DISM3S) was significantly (*p* < 0.001) associated with a broad array of microbial functions, such as nitrification (including aerobic nitrite oxidation, Spearman's rho = 0.57), cellulolysis (0.55), ureolysis (0.52), as well as aromatic compound degradation, denitrification, and anoxygenic photoautotrophy (0.43). Higher dissolved oxygen (WAT_DISS_OXYGEN_MGL) was associated with higher abundance of nitrifying (including aerobic nitrite oxidizing) and anoxygenic photoautotrophic bacteria (*p* < 0.01). Water temperature showed weak to moderate negative correlations with the abundance of bacteria involved in denitrification, fermentation, ureolysis, and anoxygenic photoautotrophy (including anoxygenic photoautotrophy S oxidizing**;** 0.43 < Spearman's rho < −0.31, *p* < 0.01). Ammonia, nitrate, total nitrogen, reactive phosphorus, water conductivity, and turbidity did not exhibit strong correlations with the abundance of most functional bacterial groups, with the exception of nitrate concentration and nitrogen-fixing bacteria (Spearman's rho = 0.37, *p* = 0.0015).

**Figure 6:**
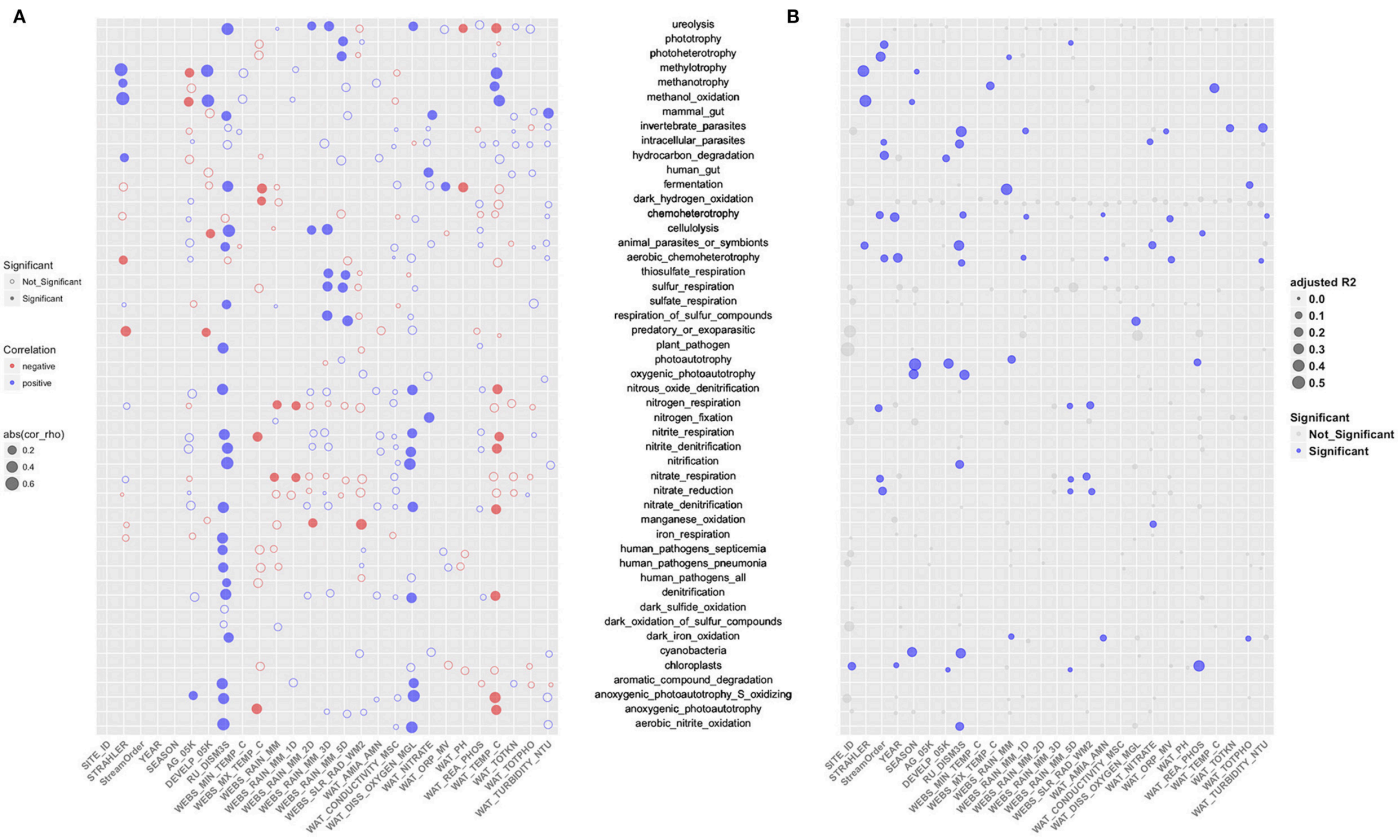
**(A)** The correlations between the relative abundance of 49 functional groups and the environmental/land use variables. Each circle represents the association between a selected variable and a particular functional group, with the size representing the strength of correlation (absolute value of spearman's rho), red color a negative correlation, and blue color a positive correlation. Solid dots show significant (*p* ≤ 0.05) correlation and circles indicate the correlation was not significant. **(B)** The variance of the compositional structure of each functional bacterial group explained by each environmental/land use variable. Each solid dot represents the community variance of a functional group explained by the variable, with blue color representing significant impact (*p* ≤ 0.05).

Based on partial RDA (Figure 6B), the compositional variance of each bacterial functional group explained by water attributes (adjusted *R*^2^) was 0.13 ± 0.01 (mean ± SE), suggesting further exploratory work is necessary. The compositional structure of the methylotrophic community (including methanol oxidizing bacteria) was influenced mostly by stream order (adjusted *R*^2^ = 0.37, *p* ≤ 0.01), while that of nitrifying bacteria, invertebrate and animal parasites (e.g., *Rickettsiella* spp.), cyanobacteria (e.g., YS2 and *Phormidium* spp.), and oxygenic photoautotrophs (e.g., *Rhodoplanes* spp.) were associated most strongly with river discharge (0.26 < adjusted *R*^2^ < 0.31, *p* < 0.05) (Figure 6B). Although the overall abundance of the ureolytic community was affected by river discharge, precipitation, dissolved oxygen, water pH and temperature, none of these variables contributed significantly to variance reductions in the compositional structure of this functional group (small gray dots in Figure 6B).

Overall, hydrological and physiochemical attributes of water courses were not strongly associated with the compositional structure of functional communities. OTUs assigned as human pathogens (e.g., *Staphylococcus*), nitrifiers (e.g., *Nitrospira* and other *Nitrospiraceae* spp.), and fermenters (e.g., *Propionibacterium*) were more abundant in spring in agricultural ditches, while samples from higher order streams and/or water courses with more mixed upstream land-uses were rich in potential hydrocarbon degrading (e.g., *Methylocystaceae* spp.), photoheterotrophic (e.g., *Rhodobacter* spp.), and methylotrophic (e.g., *Methylophilaceae* spp.) bacteria.

### Impact of land uses on bacterial community network

**Figure 7:**
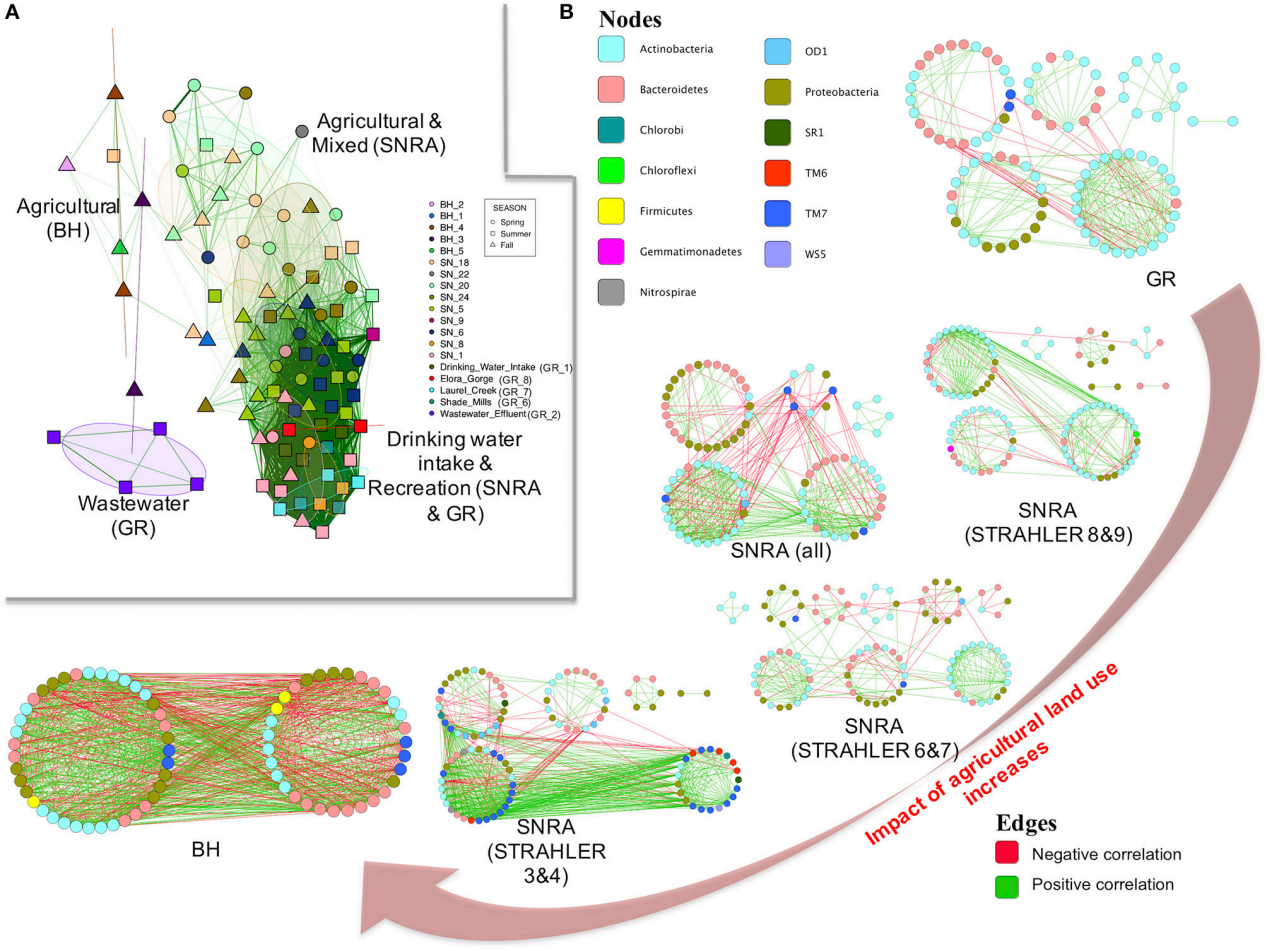
**(A)** A network showing similarity of the bacterial communities recovered at each sampling site. Each symbol represents one sample. Samples sharing more than 30% community similarity were connected by green edges and were placed closer to each other; the darker the edges, the higher the community resemblance. The overlaid circles are 50% confidence regions of ellipses of samples from each sampling site. **(B)** Ecological association networks of 97%-identity OTUs at SNRA, BH, and GR based on RMT-approach. The size of each node (OTU) is proportional to the degree (linked edges). The color of each node was determined by phylum. The green edges represent positive correlations between two nodes, while red edges represent negative correlations. The network plots are arranged by the proportion of agricultural land uses around the sampling watersheds.

To obtain a generic understanding of how land use and water quality influence aquatic microbial community compositional structure and interactions among community members, a limited number of water samples were retrieved from BH in fall 2012 (a sampling time when manures are often applied to land) and at GR in summer 2011 (a time period for recreational activities in water). Overall, bacterial communities had the highest alpha (*p* < 0.001) and beta-diversity (ANOSIM R = 0.91, *p* < 0.001) at BH sites (Supplementary Figures S7, S8) and the lowest diversity at GR (Table 2). The network plot in Figure 7A shows the divergence of the community composition among samples. The Figure suggests that the bacterial community of agriculturally dominated sampling sites (at both SNRA and BH) had < 30% similarity (lack of connections between samples), while those of samples from main river stems at SNRA and GR displayed higher compositional resemblance (dark green edges between samples). The bacterial communities from site GR_2 downstream of the wastewater effluent had the most unique compositional structure (Supplementary Figure S8). The shared and unique OTUs found at SNRA, GR, and BH are shown in Supplementary Figure S8D. The bacterial families differed in abundance between SNRA and BH samples collected from fall 2012 are shown in Supplementary Figure S9.

Based on the 16S rDNA metabarcodes in this study, fecal indicators (fecal coliform) and pathogens (e.g., *Salmonella, Campylobacter*) were either not recovered, or were detected at low levels (Supplementary Figure S10). However, both the functional and taxonomic profiles of the wastewater effluent impacted site (GR_2) were distinct from other water samples, with elevated amounts of animal parasites or symbionts (e.g., *Roseburia, Fibrobacter, Clostridium, Coprococcus, Bacteroides*, and *Prevotella*) (Supplementary Figure S8). Furthermore, compared with drinking water intake samples, GR_2 samples were rich in *Alphaproteobacteria, Clostridia, Bacteroidia, Fusobacteriia*, and *Bacilli*, but with reduced prevalence of *Flavobacteriia* (Supplementary Figure S8B). The distribution of bacterial genera containing members of naturally occurring fecal indicators and waterborne-/fecal pathogens recovered by 16S rDNA metabarcodes in this study is shown in Supplementary Figure S10.

Using the Spearman's rank correlation coefficient matrix and RMT-based network analysis, the MENs of bacterial OTUs were constructed and compared among communities at BH, SNRA (overall), SNRA STRAHLER = 3&4, SNRA STRAHLER = 6&7, SNRA STRAHLER = 8&9, and GR. Much lower similarity thresholds were selected by RMT for BH (Spearmen's rho = 0.31), SNRA overall (0.55), and SNRA STRAHLER 3&4 (0.57), in relation to those of SNRA STRAHLER 6&7, SNRA STRAHLER = 8&9, and GR (0.63–0.79) (Table 3). Therefore, more community members were included when constructing the empirical networks for BH and SNRA STRAHLER = 3&4 samples. The topological properties of all MENs are shown in Table 3, which suggests networks of agriculturally dominated sites (BH, SNRA STRAHLER = 3&4) had higher connectivity, represented by a higher number of links (number of edges), shorter path length (geodesic distance), and a higher clustering coefficient (high density of connections). MENs of BH and SNRA STRAHLER = 3&4 also exhibited a lower degree of modularity, i.e., higher connection between modules, and poorly fitted with power-law models (*R*^2^ < 0.54) than MENs under less “direct” agricultural association (*R*^2^ > 0.72). By comparing the empirical and corresponding random MENs, all networks in the current study displayed typical small-world characteristics (short path and high clustering coefficient). In addition, MENs of larger watercourses (GR and SNRA STRAHLER = 8&9) contained mainly *Actinobacteria, Bacterioidetes, Proteobacteria*, and TM7, while at smaller agricultural drainage ditches, MENs contained additional taxonomic lineages such as TM6 and OD1.

**Table 3:** Topological properties of molecular ecological networks (MENs) of microbial communities.

			**SNRA**				
**Network**	**Index**	**BH**	**ALL**	**STRAHLER 3&4**	**STRAHLER 6&7**	**STRAHLER 8&9**	**GR**
	Sample size	7	71	30	28	13	16
	Number of original OTUs	82	471	271	174	148	158
	Similarity threshold (Spearman's rho)	0.31	0.55	0.57	0.63	0.79	0.76
	Number of nodes	70	91	103	101	103	95
	Number of edges	1247	396	629	255	297	268
	R square of power-law						
	Power-law (free-scale)	–	0.439	0.498	0.542	0.825	0.745
	Truncated power-law (broad scale)	–	0.343	0.013	0.824	0.723	0.796
	Exponential (faster-decaying)	–	0.544	0.542	0.786	0.844	0.819
Empirical	Modularity (no. of modules)	0.091 (2)	0.377 (5)	0.256 (6)	0.673 (9)	0.532 (8)	0.537 (6)
	Average clustering coefficient (C)	0.562	0.485	0.461	0.468	0.359	0.399
	Harmonic geodesic distance (L)	1.319	2.47	2.218	3.194	2.876	3.061
Random	Average modularity ± SD	0.057 ± 0.025	0.233 ± 0.009	0.168 ± 0.005	0.381 ± 0.011	0.335 ± 0.009	0.333 ± 0.011
	Average clustering coefficient (Cr) ± SD	0.522 ± 0.002	0.238 ± 0.020	0.367 ± 0.021	0.080 ± 0.016	0.111 ± 0.014	0.126 ± 0.018
	Harmonic geodesic distance (Lr) ± SD	1.319 ± 0.000	2.124 ± 0.014	2.019 ± 0.014	2.622 ± 0.026	2.487 ± 0.026	2.472 ± 0.028

## Discussion

### Key environmental determinants of bacterial community diversity and assemblage

Land uses due to agricultural and other human activities can influence the function and health of freshwater ecosystems, as demonstrated by many studies and reviews (Guber et al., 2006; Delpla et al., 2011; Jung et al., 2014; Cho et al., 2016). Linkages between microbial diversity/function and environmental stressors/drivers are critical for supporting the development of robust indicators of aquatic ecosystem health and resilience (Llirós et al., 2014; Battin et al., 2016). The current study suggests that at SNRA globally, the diversity of the bacterial community was, on average, higher in lower order streams (primarily agriculturally dominated drainage ditch systems) relative to larger watercourses. The water sampling sites for the lowest stream order systems were closer to the bed of the stream and instream biota, as well as closer to adjacent stream banks relative to the deeper and wider larger order systems sampled (small stream/ditches can be only meters wide). Due to these factors, changes in the aquatic microbiome in the small stream/ditch waters will likely be more sensitive to drainage, rainfall, and runoff events than the sampled water column of the larger systems examined (Frey et al., 2015; Read et al., 2015; Dong et al., 2016; Hosen et al., 2017; Langenheder et al., 2017). Thus, the smaller water courses will likely experience more amplified and abrupt pulses and thrusts of soil bacteria from adjacent terrestrial features (e.g., fields) during transport events, as per the two agriculturally dominated sites (Figure 2, Supplementary Figure S5) during a large summer rainfall event that occurred in the SNRA sampling region, of which microbes of soil origin consisted of one-fifth of the overall community (Supplementary Figure S5). For smaller stream systems, environmental sorting was also more influential, as reflected by the stronger association between the diversity, compositional, and/or functional structure and environmental stressors/drivers. Bacterial communities from larger watercourses had higher compositional similarity, in spite of being impacted cumulatively by a more diverse array of upstream land uses and water pollution drivers (Figure 7A). Although the generalized global relationship between diversity and stream order was inverse, CART analyses indicated that during common periods of low to no rain and water flow (i.e., hydrologically inactive periods), average diversity was “relatively” higher in larger order streams, relative to smaller ones. This suggests data mining analytics that identify “local” contextualized interactions will be critical for uncovering the factors that shape microbiome diversity and structure in complex systems like watersheds.

As reviewed by Zeglin (2015), most studies investigating riverine ecosystems in response to environmental factors found hydrology, land use, and water abiotic properties (e.g., temperature, metals, and micronutrients levels) had most significant impact on aquatic microbial communities. For example, land use or landscape changes, as represented by percent impervious cover (% IC) (Wang et al., 2011), or seepage vs. drainage lakes (Yannarell and Triplett, 2005), were found to be important factors in predicting freshwater bacterial communities associated with denitrification (Wang et al., 2011). Montuelle et al. (2010) found benthic microbial communities were sensitive to rainfall intensity and changes in metal and nutrient concentrations of the water. It is noteworthy that some studies did not document evidence of changes in water microbiome under the influences of agricultural activities and urban runoff. Instead, water temperature and river flow rate, partially due to seasonal weather changes, were important determinants with respect to the temporal patterns in community structure (Stepanauskas et al., 2003; Crump and Hobbie, 2005; Ibekwe et al., 2013), as also suggested in this current study.

Agricultural drainage and runoff can carry a wide range of agricultural chemicals (Smith et al., 1993) that can potentially affect bacterial community composition, transiently, or permanently (Montuelle et al., 2010; Zeglin, 2015). In this study, we showed that agriculturally dominated watershed sites (SN_18, 20, 22) had higher specific conductivity, turbidity, and nutrients (e.g., nitrogen), but were lower in dissolved oxygen, relative to SN_24 (reference site) and SN_8 (larger watercourse site) (Supplementary Figure S1). Spietz et al. (2015) noted that bacteria can undergo significant changes well before dissolved oxygen levels reach hypoxia thresholds, indicating their usefulness as early warning indicators of oxygen-stressed aquatic ecosystems, e.g., under progressive eutrophic conditions (Ansari et al., 2011). Bernhard et al. (2005) found that increased salt concentrations resulted in the loss of diversity of the ammonia-oxidizing bacteria communities, which was not specifically identified in the current study. However, we did demonstrate strong positive associations among nitrate content and nitrifying communities (mainly consisted of *Nitrospiraceae* spp., and *Nitrospirae* spp.) (Figure 6).

In this study, the denitrifying communities (mainly consist of *Rhodoplanes* spp., *Acidovorax* spp., and *Paracoccus* spp.), which can be inhibited by higher oxygen concentrations (Gómez et al., 2002; Tan et al., 2013), were positively correlated with dissolved oxygen levels (*R* = 0.36, *p* = 0.002). Early studies have found facultative anaerobic members in all three genera that are capable of complete denitrification even under 90–100% air saturation (Hiraishi and Ueda, 1994; Neef et al., 1996; Michotey and Bonin, 1997; Huang et al., 2012). In addition, water particles that harbor sub-oxic or even anoxic microenvironments may facilitate anaerobic microbial activity (e.g., denitrification, sulfate reduction, and fermentation), as observed in marine ecosystems (Michotey and Bonin, 1997; Dang and Lovell, 2016). Also all correlation analyses relating functional groups with water physiochemical properties in natural flowing systems require some degree of conservatism when assessing cause and effect. Water properties that may be associated with the function and proliferation of certain microbes may not necessarily be present when such microbes are observed in the water column. Regarding the positive correlations among denitrifiers and dissolved oxygen found herein as an example, detection of resident denitrifiers in the water could also take place under the influence of fresh well-oxygenated rain and drainage water that stimulated and sustained mobilization and transport of these bacteria from otherwise anaerobic compartments of the watershed during a storm or flow event. In fact, Frey et al. (2015) reported that at the same SNRA sampling sites of this study, anaerobic conditions favorable for denitrifying bacteria were found within a few centimeters below the surface of exposed streambed sediments, and, moreover, resident bacteria in these shallow sediments could be easily transported by rainfall/runoff events to adjacent streams.

The lower pH at the agriculturally impacted sites (Supplementary Figure S1) may have resulted from fertilizer inputs. We also noticed a decline in ACK-M1 prevalence at agricultural watersheds (*R* = 0.67, *p* < 0.001) (Figure 4), although Methé and Zehr (1999) observed an enrichment of this taxon group in acid stressed lakes with monomeric and non-labile aluminum. We also observed negative correlations between urban development patterns and pathogens/animal parasites, which may be interpreted as positive associations with agricultural land uses (e.g., fertilization with manure or compost**;** Guber et al., 2006; Cho et al., 2016) in the SNRA (Wilkes et al., 2013b). Therefore, the environmental gradients associated with pollution/impact source (diffuse or point pollution) should, ideally, be considered in analyses assessing the biogeography of bacterial communities.

### Enrichment of functional bacterial diversity under the impact of agricultural land uses

Members of *Actinobacteria, Proteobacteria*, and *Firmicutes* contributed to almost all recovered functionalities in the current study, showing broad functional coverage and importance. Despite the fact that *Actinobacteria* was most abundant in these watersheds, as also observed by Zeglin (2015), the richness of *Proteobacteria* and *Firmicutes* were higher than that of *Actinobacteria*. Such less abundant but more diversified taxonomic lineages, termed as rare-biosphere, were found to be highly active (Lawson et al., 2015) especially under unexpected environmental perturbations (Wang et al., 2017).

Functional redundancy is referred to as “taxonomically distinct species that exhibit similar ecological functions” (Guillemot et al., 2011). Across all sampling sites, time points, and locations, chemoheterotrophs (including aerobic chemoheterotrophs) were most abundant in surface water, with taxonomic lineages across multiple bacterial phyla. However, close to 50% of the OTUs associated with chemoheterotrophy were found unique at a variety of stream orders. The ubiquity and cosmopolitan nature of chemoheterotrophs highlights the importance of carbon assimilation (Nakagawa and Takai, 2008) in freshwater ecosystems. We found nitrifiers were significantly more abundant at agriculturally impacted sites, while denitrifiers were significantly more abundant in larger order streams that receive waters from a variety of sources, including the agricultural drainage networks. Outside of the aquatic ecosystem, Banerjee et al. (2016) and Horemans et al. (2016) observed the enrichment of keystone microbes in nutrients or linuron herbicide amended soils, suggesting functional redundancy in C decomposition or linuron mineralization in agricultural fields. Higher redundancy in a functional group (e.g., N or C cycling), therefore, may imply stronger species selection along specific environmental gradients (e.g., nutrient levels) (Miki et al., 2014).

Miki et al. (2014) described and quantified a positive association between the microbial species richness (diversity) and the community multifunctionality (represented by ortholog richness) redundancy. For the recreation and drinking water intake sites (GR sites and SN_1&8), the lower average diversity and the lack of intrinsic functional redundancy for many chemicals/pollutants (e.g., agricultural P/N pressures) may insinuate their intolerance to a broader suite of singular and multiple environmental stressors, and slower responses to transient environmental pulses/changes in such water systems. In addition, we observed a negative correlation between pH and ureolytic bacteria abundance, suggesting that higher pH in the larger watercourses (with lower diversity) may slow down the degradation of some herbicides in the sulfonyl urea family (Furmidge and Osgerby, 1967). Although speculation, such systems therefore may be vulnerable to environmental and anthropogenic stressors, and may have a reduced capacity to “self-purify” polluted waters when transient pollution occurs. Overall, from a purely conceptual perspective, redundancy in microbial functionality could be critical for maintaining biogeochemical processes essential for healthy aquatic ecosystem function under system stress (Tully et al., 2017), as observed in some soil environments (Yin et al., 2000).

### Community dynamics and ecological networks revealed land use impacts and degree of contamination

Characterizing the bacterial community can facilitate tracking the origins and sources of microbes in the water system (Henry et al., 2016; McCarthy et al., 2017). This information can be invaluable in diagnosing sources of pollution and in developing mitigation approaches at the watershed level (Wilkes et al., 2013a, 2014). Examining the functionality of the bacterial community can also serve as an indicator for stream health and stress (Segovia et al., 2016).

It is well known, however, that 16S rDNA markers have low discriminating power for species-level classification (Rajendhran and Gunasekaran, 2011; Poretsky et al., 2014), while universal primers used for amplicon-based HTS may lack taxonomic coverage (Fischer et al., 2016). Thus, it is difficult using 16S rDNA metabarcodes for microbial source tracking quantitatively and accurately, especially at species/subspecies levels. Indeed, while many enteric pathogens were previously found to be relatively abundant using alternative molecular diagnostic assays in the same watersheds (Wilkes et al., 2011), the current study did not detect, or detected at very low levels, fecal bacteria.

Our study suggests that when using metabarcoding approach, bacterial community structure, rather than individual taxonomic lineage, is potentially a better predictor of bacterial sources and mobilization drivers in these watershed settings. By comparing the taxonomic and functional profiles, as well as by calculating the community similarity among sampling units originating from different water sources and locations, we were able to identify sampling sites and/or sampling time points during which transport/mobilization/differentiated pollution events likely occurred. As an example, drinking water intake sites from GR and SNRA had similar community profiles, but differed substantially from wastewater effluent impacted sites and the more intensive agricultural sites in BH (Figure 7A).

As mentioned previously, RMT is a more objective approach for selecting correlation coefficient thresholds when constructing MENs (Luo et al., 2006; Zhou et al., 2010, 2011; Deng et al., 2012). Empirical evidence in our studies suggests that compared with the cutoff determined by RMT, the use of a larger threshold resulted in smaller networks (more nodes and linkages being removed) with higher modularity (Supplementary Figures 12A,C and Supplementary Table S2), while a smaller threshold resulted in larger networks with strongly correlated modules being disguised by too many retained interconnections (edges) (Supplementary Figures 12B,D and Supplementary Table S2). Although different agricultural production systems would induce intricate changes in both functional and taxonomic compositional structures of aquatic bacterial communities, the ecological networks (i.e., coexisting association) of community members at agricultural watersheds (Figure 7B) all displayed typical “small-world” characteristics, high connectivity, low degree of modularity, and did not fit well with power-law degree distributions which was somewhat disparate with the topology of microbial networks recovered from other aquatic (Steele et al., 2011; Kara et al., 2013; Hu et al., 2014) and soil (Barberán et al., 2012) ecosystems. Furthermore, MENs at agriculturally dominated sites (BH and lower order streams of SNRA) shared minimum number of nodes (OTUs), compared with those at other sites (Supplementary Figure S11). Such rather drastic changes in coexisting patterns revealed by MENs suggest a highly sensitive and perhaps more transient bacterial community under the elevated influence of agricultural land use activities.

## Conclusions

The current study investigated bacterial communities in freshwater from a suite of mixed-use but predominately agricultural watersheds. Some core findings are highlighted below:

- Globally speaking, community diversity was somewhat higher at smaller agriculturally dominated drainage ditch/small stream systems, compared to larger stream order systems. However, data mining revealed locally context dependent relationships/interactions suggesting that for common lower to no-flow and lower relative accumulated rainfall situations, higher diversity was observed at the larger order streams, in relation to smaller order systems.
- The variance of both the diversity and community composition of aquatic bacterial communities was best explained by stream order and stream discharge. Other environmental variables had a much smaller or insignificant contribution to explained variance. Stream discharge was significantly (all positively) correlated with many bacteria functional groups.
- The changes in bacterial community compositional structure were more volatile in the lower order agriculturally dominated creeks and drainage ditches relative to larger order river systems. Incident bacteria mobilized by hydrological events were detected more readily in the smaller watercourses where the juxtaposition (and hydrological connectivity) of the sampled water column to stream and near-stream mineral and organic substrates and the adjacent terrestrial environment, was greater.
- Discrepancies among cause and effect between functional groups and water physiochemical properties found in the literature could, in part, be related to microbes in water samples not necessarily reflecting the nature of the environmental conditions at which the functional targets existed and/or thrived in the environment. Therefore, ascribing biophysical cause and effect among bacteria function and water physiochemical disposition in sampled water needs to be exercised with caution, especially for flowing systems.
- When using metabarcoding approach, bacterial community structure, rather than individual taxonomic lineage, appears to be a better predictor of bacterial sources in watershed settings. Comparison of the taxonomic and functional profiles and community similarity structure via network analyses allowed for the identification of water pollution sources and degree of agricultural and urban land use intensity.

## Author contributions

WC processed and analyzed the metabarcoding data. DL and GW performed the CART analyses. WC, DL, and GW drafted the manuscript. KP, JT, and IK helped edit the manuscript. DL, GW, IK, KP, JT, JC, and CAL performed field sampling and laboratory experiments, assimilated the data, and contributed to writing the materials and methods. All co-authors reviewed the manuscript.

### Conflict of interest statement

The authors declare that the research was conducted in the absence of any commercial or financial relationships that could be construed as a potential conflict of interest.
