# Supplementary material for: Aquatic Bacterial Communities Associated With Land Use and Environmental Factors in Agricultural Landscapes Using a Metabarcoding Approach
Source: Front Microbiol. 2018 Oct 30;9:2301. doi: 10.3389/fmicb.2018.02301 (PMC6218688; doi:10.3389/fmicb.2018.02301)
Supplement: Supplementary file 3 [file Presentation_1.pdf]

Supplementary Material

Aquatic bacterial community associated with land use and environmental factors in agricultural landscapes using a metabarcoding approach

Wen Chen[1*], Graham Wilkes[1], Izhar U.H. Khan[1], Katarina D.M. Pintar[2], Janis L. Thomas[3], C. André Lévesque[1], Julie T. Chapados[1], Edward Topp[4] and David R. Lapen[1]

*** Correspondence:** Wen Chen: Wen.Chen@AGR.GC.CA;

1 Supplementary Figures and Tables

1.1 Supplementary Figures

Supplementary Figure S1. Partial least squares discriminant analysis (PLS-DA) for classification of SNRA watersheds based on land use, hydrology of streams and water physiochemical properties. The ellipses enclose samples within 1 standard deviation of means from A) each sampling site; and B) each sampling season at B1) SN_18; B2) SN_5; B3) SN_24; B4) SN_6.

Supplementary Figure S2. The number of A) shared and unique OTUs and B) functional groups was found the most in watersheds of lower stream orders (STRAHLER=3,4), and the least in watersheds of higher stream orders (STRAHLER= 8,9). Sites of higher stream orders shared 87% OTUs with sites of lower and medium stream orders, while sites of lower stream orders only shared 56% OTUs with those of higher and medium stream orders. C) Species accumulation curve for each SNRA site shows that the species richness was highest at SN_18 and 20 (of low stream orders).

Supplementary Figure S3. Permutation test for homogeneity of multivariate dispersions of SNRA samples within each stream order grouping (low= STRAHLER 3,4; medium = STRAHLER 5,6,7; high= STRAHLER 8,9): A) heterogeneous dispersions among communities within each stream order

grouping; B) the distance of beta diversity to the centroid within each stream order grouping. The largest distances to the centroid were observed at sites of lower stream orders, and smallest distances at sites of higher stream orders.

Supplementary Figure S4. The ANOSIM results show that there were no significant differences in the bacterial community compositional structures among SNRA samples from A) different seasons ($R = 0.088$), or from B) different sampling years ($R = 0.074$), despite possessing p-values < 0.05.

Supplementary Figure S5. Proportion of microbes from different environments in water samples collected on A) 23-August-2010 with the highest diversity at SN_18&20 (SW-TD=893 and 263, respectively); B) 06-June-2011 with the lowest diversity at SN_18&20 (SW-TD=3 and 2, respectively); C) 29-August-2011 with almost the highest diversity at SN_1&8 (SW-TD=43 and 35, respectively). The dark brown represents soil-associated bacteria. D) Summary of diversity, cumulative rainfall and the proportion of soil-associated bacteria based on BLAST results against MetaMetaDB for each sample in A), B) and C).

Supplementary Figure S6. The existence and average relative abundance of OTUs in functional bacterial groups involved in A) cellulolysis; B) nitrification; C) respiration of sulfur, D) fermentation (OTU labels were removed), or considered as E) animal parasites or symbionts.

Supplementary Figure S7. Water samples collected at BH and SNRA from fall 2012: A) shared and unique OTUs at BH and SNRA; B) Relative abundance of bacterial classes in each sample; C) heatmap of relative abundance of OTUs (> 30 reads) belonging to at least one functional group, using the double square roots of relative abundance matrix.

Supplementary Figure S8. A, B&C) Water samples collected at GR and SNRA from summer 2011: A) relative abundance of bacterial functional groups in each sample; B) relative abundance of bacterial functional groups at the class level; C) heterogeneous dispersion of bacterial functional

groups using hellinger transformed abundance data. D) Shared and unique OTUs found at BH, SNRA and GR (including all samples).

Supplementary Figure S9. Bacterial families differed significantly in relative abundance between the samples collected at BH and SNRA during fall 2012.

Supplementary Figure S10. Distribution of selected bacterial genera containing fecal indicators.

Supplementary Figure S11. Pairwise network comparison: A) shared nodes (white) between the molecular ecological networks (MENs) of GR and SNRA STRAHLER=8&9 (drinking or recreational water) samples; red nodes are only in GR MEN while green nodes are only in SNRA STRAHLER= 8&9 MEN; B) shared nodes (white) between MENs of SNRA STRAHLER = 3&4 and BH (agriculturally dominated watersheds) samples; red nodes are only in BH MEN while green nodes are only in SNRA STRAHLER= 3&4 MEN.

Supplementary Figure S12. Molecular ecological networks (MENs) constructed using arbitrary (A&C, Spearman's rho = 0.60) and RMT-based (B&D, Spearman's rho = 0.31 for B, 0.79 for D) selection of correlation coefficients thresholds. A&B) MENs of BH samples. C&D) MENs of SNRA STRAHLER=8&9 samples. Nodes are colored by phyla; positive and negative correlations are in green and red, respectively.

1.2 Supplementary Tables

Supplementary Table S1. Water samples and associated Sequence Reading Archive (SRA) accession numbers used in the current study.

Supplementary Table S2. Global network properties when using correlation coefficient thresholds arbitrarily selected or selected by RMT-based method.

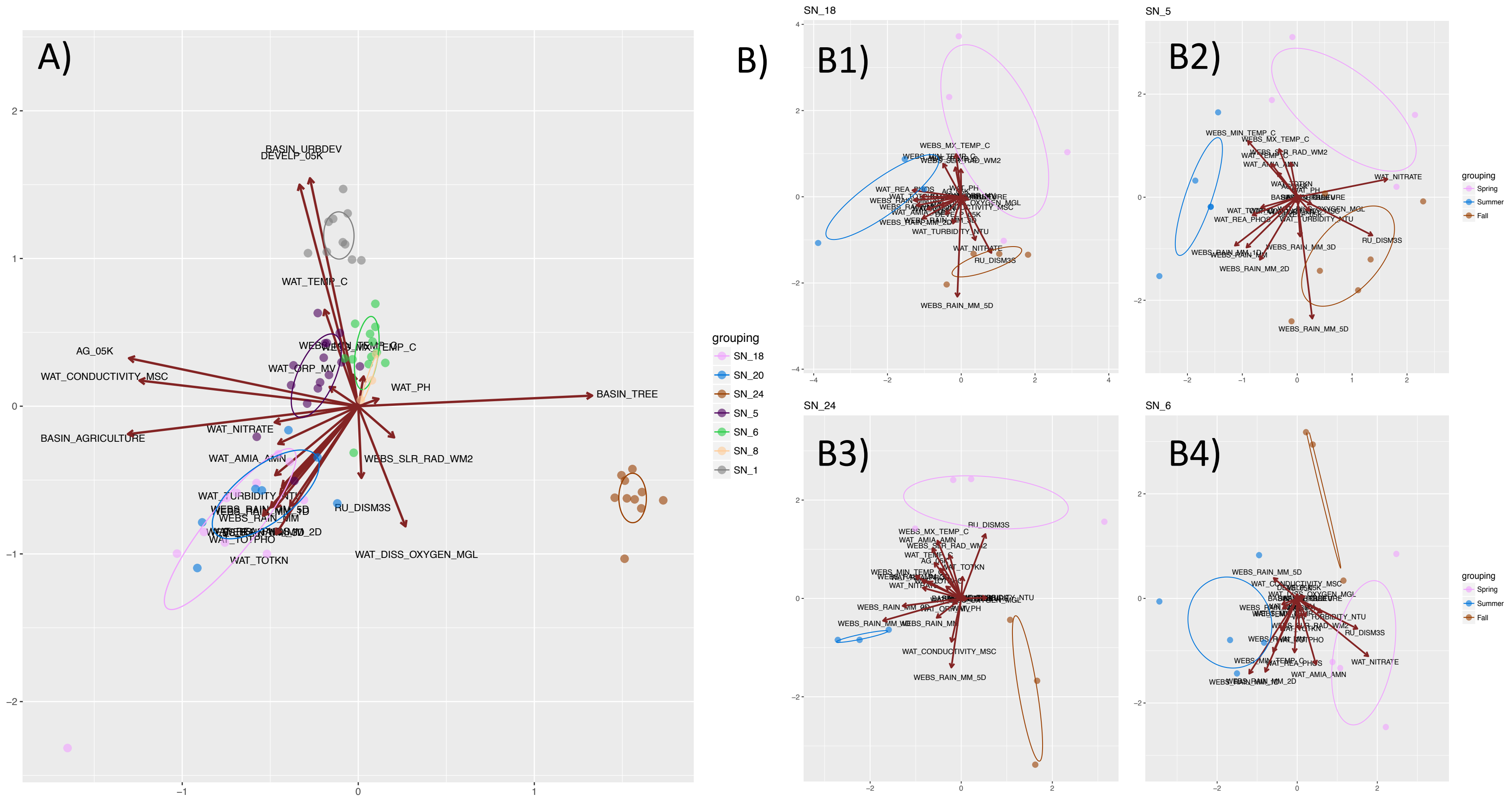

Supplementary Figure S1. Partial least squares discriminant analysis (PLS-DA) for classification of SNRA watersheds based on land use, hydrology of streams and water physiochemical properties. The ellipses enclose samples within 1 standard deviation of means from A) each sampling site; and B) each sampling season at B1) SN_18; B2) SN_5; B3) SN_24; B4) SN_6.

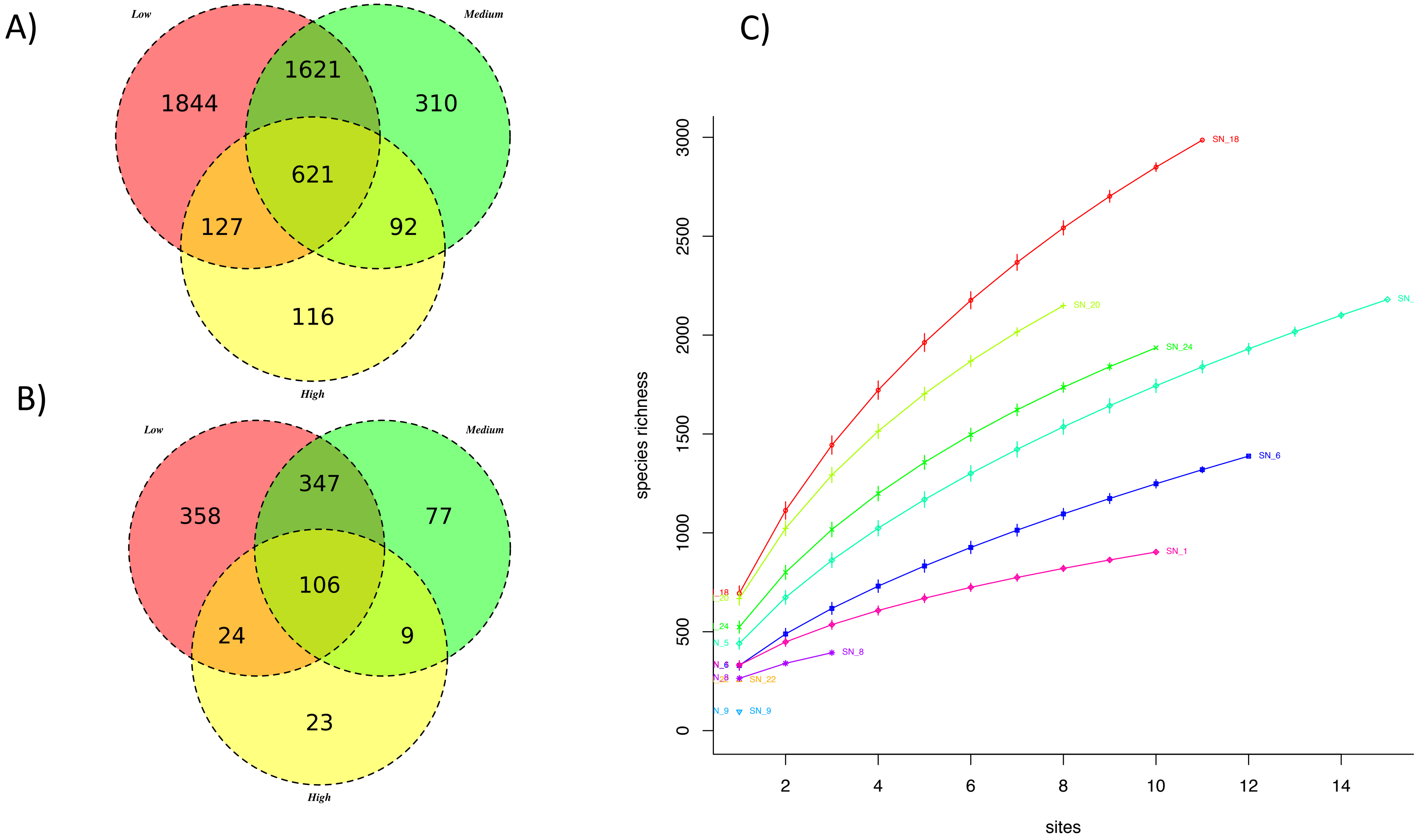

Supplementary Figure S2. The number of A) shared and unique OTUs and B) functional groups was found the most in watersheds of lower stream orders (STRAHLER=3,4), and the least in watersheds of higher stream orders (STRAHLER= 8,9). Sites of higher stream orders shared 87% OTUs with sites of lower and medium stream orders, while sites of lower stream orders only shared 56% OTUs with those of higher and medium stream orders. C) Species accumulation curve for each SNRA site shows that the species richness was highest at SN_18 and 20 (of low stream orders).

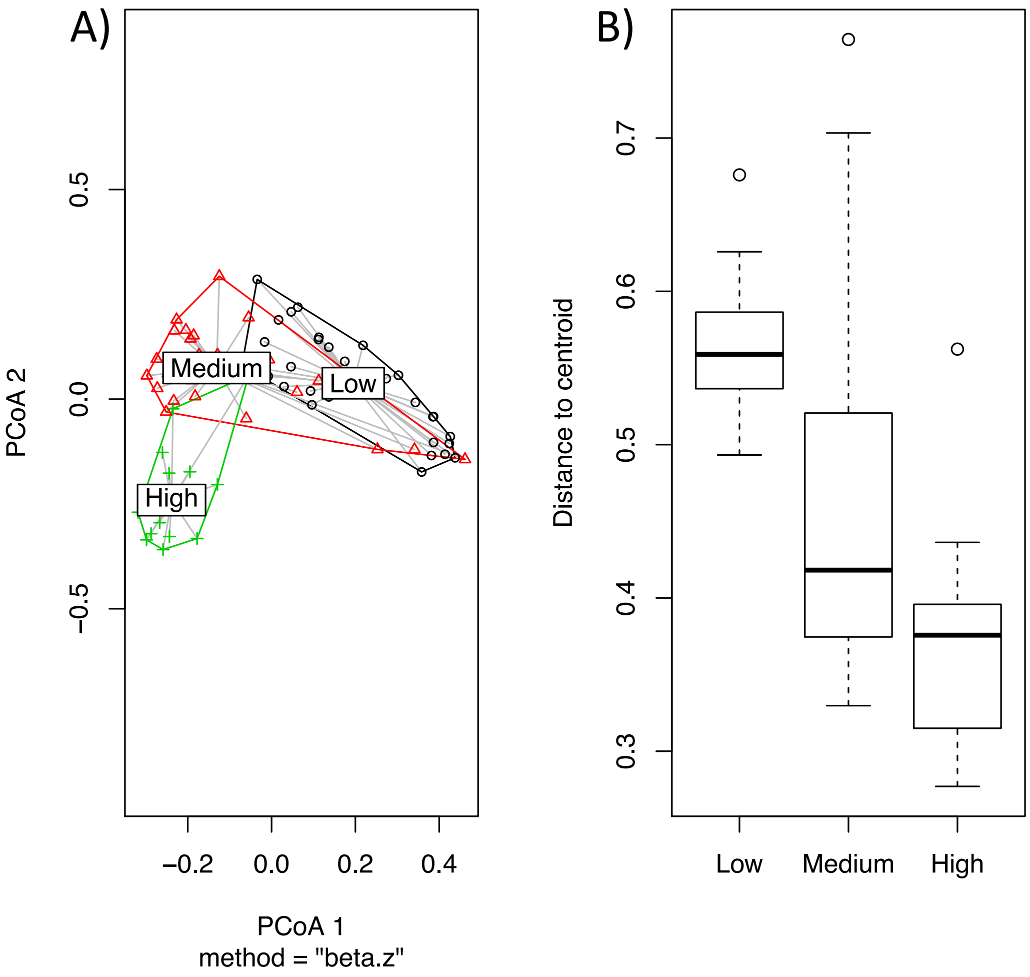

Supplementary Figure S3. Permutation test for homogeneity of multivariate dispersions of SNRA samples within each stream order grouping (low= STRAHLER 3,4; medium = STRAHLER 5,6,7; high= STRAHLER 8,9): A) heterogeneous dispersions among communities within each stream order grouping; B) the distance of beta diversity to the centroid within each stream order grouping. The largest distances to the centroid were observed at sites of lower stream orders, and smallest distances at sites of higher stream orders.

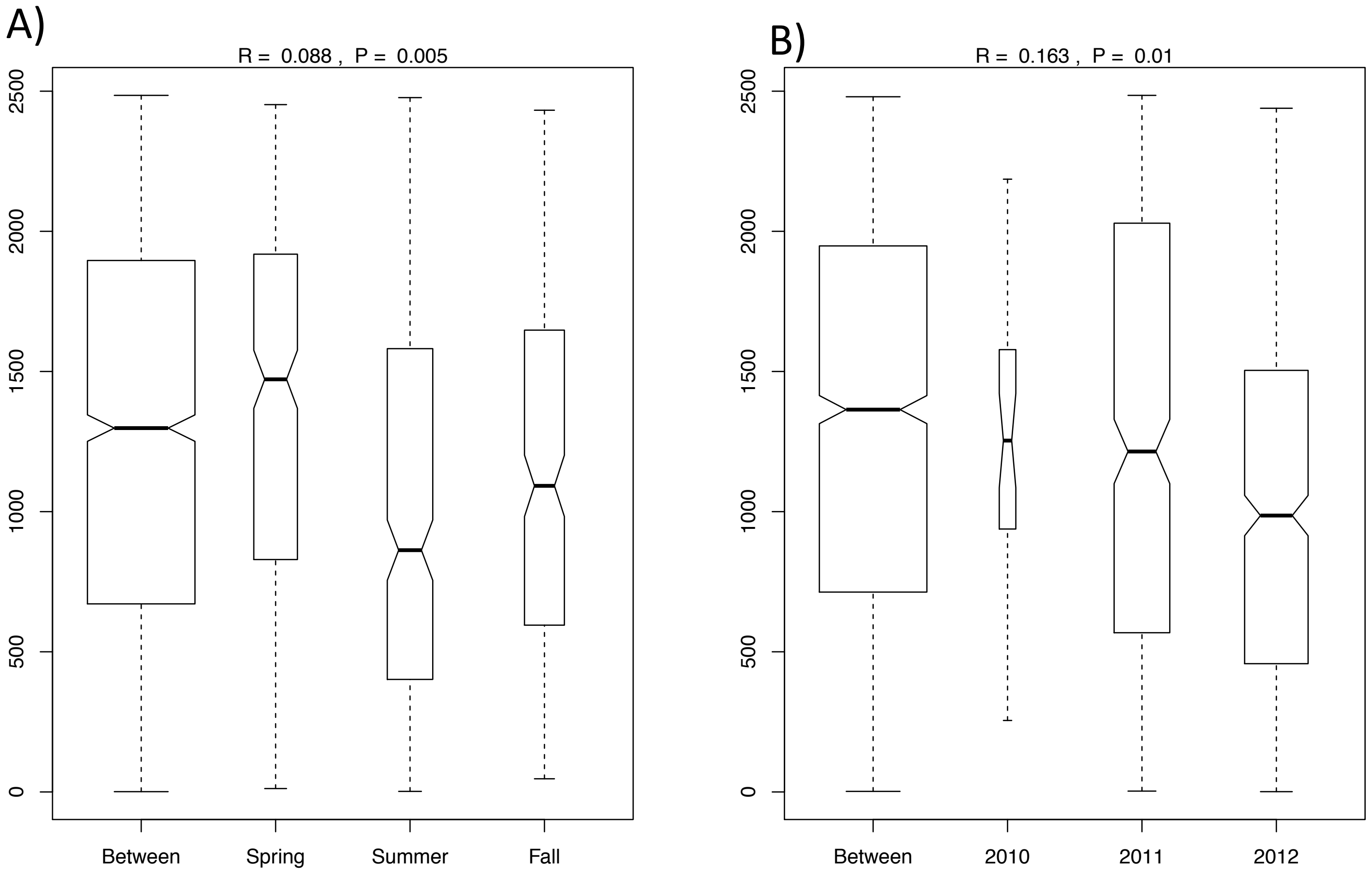

Supplementary Figure S4. The ANOSIM results show that there were no significant differences in the bacterial community compositional structures among SNRA samples from A) different seasons (R = 0.088), or from B) different sampling years (R = 0.074), despite possessing p-values < 0.05.

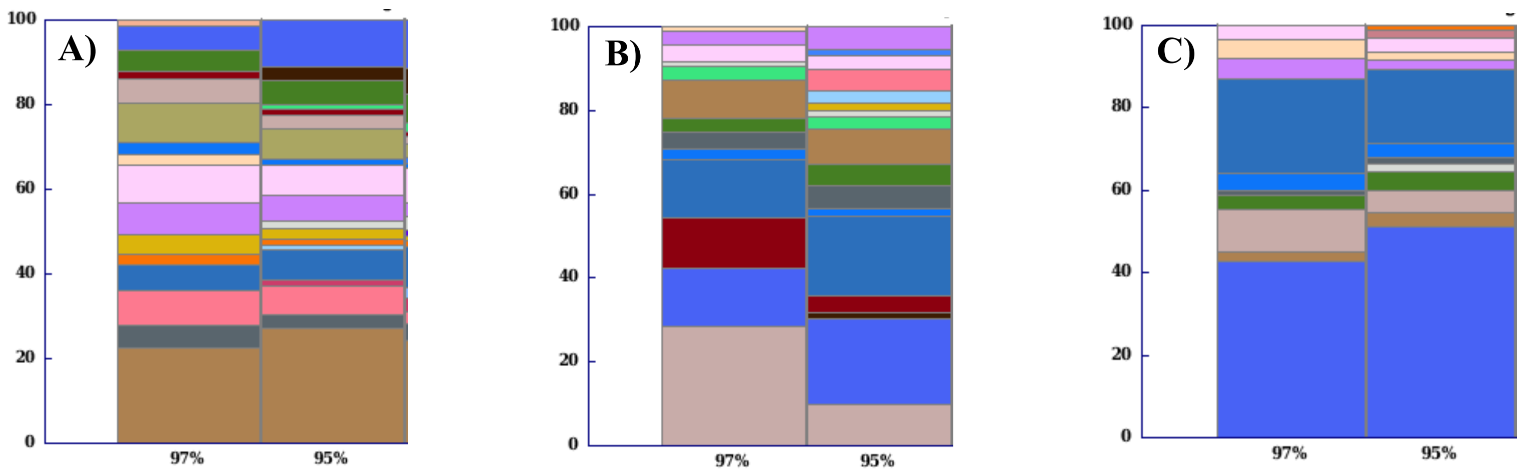

Samples MST-SNC-18-2010235 & MST-SNC-20-2010235; collected on 23-August-2010, highest diversity at SN_18&20.

Samples MST-SNC-18-2011157 & MST-SNC-20-2011157; collected on 06-June-2011, lowest diversity at SNRA_18&20.

Samples MST-SNC-1-2011241 & MST-SNC-8-2011241; collected on 29-August-2011, highest diversity at SNRA_1&8.

D)

Sample ID	SITE_ID	DATE	WEBS_RAIN _MM_5D	Diversity SW_TD	Microbes from soil habitats BLAST identity 97%	95%
MST-SNC-18-2010235	SN_18	23-08-2010	44.9	893	22.57%	26.97%
MST-SNC-20-2010235	SN_20	23-08-2010	44.9	263		
MST-SNC-18-2011157	SN_18	06-06-2011	0.1	3	9.11%	8.40%
MST-SNC-20-2011157	SN_20	06-06-2011	0.1	2		
MST-SNC-1-2011241	SN_1	29-08-2011	14.1	43	2.28%	3.41%
MST-SNC-8-2011241	SN_8	29-08-2011	14.1	35		

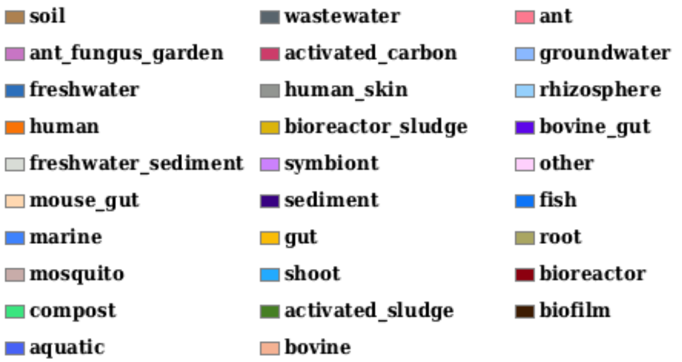

Supplementary Figure S5. Proportion of microbes from different environments in water samples collected on A) 23-August-2010 with the highest diversity at SN_18&20 (SW-TD=893 and 263, respectively); B) 06-June-2011 with the lowest diversity at SN_18&20 (SW-TD=3 and 2, respectively); C) 29-August-2011 with almost the highest diversity at SN_1&8 (SW-TD=43 and 35, respectively). The dark brown represents soil-associated bacteria. D) Summary of diversity, cumulative rainfall and the proportion of soil-associated bacteria based on BLAST results against MetaMetaDB for each sample in A), B) and C).

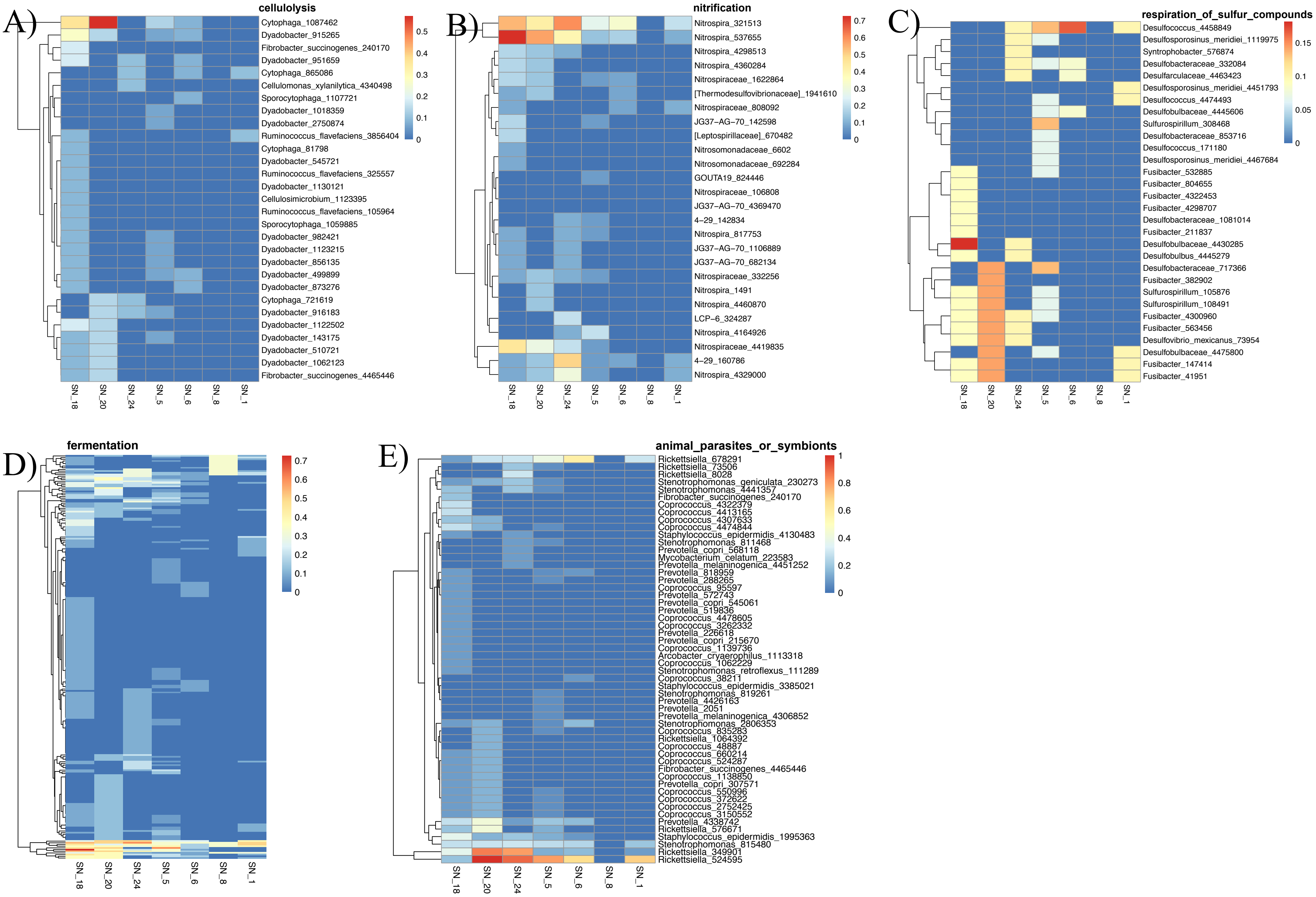

Supplementary Figure S6. The existence and average relative abundance of OTUs in functional bacterial groups involved in A) cellulolysis; B) nitrification; C) respiration of sulfur, D) fermentation (OTU labels were removed), or considered as E) animal parasites or symbionts.

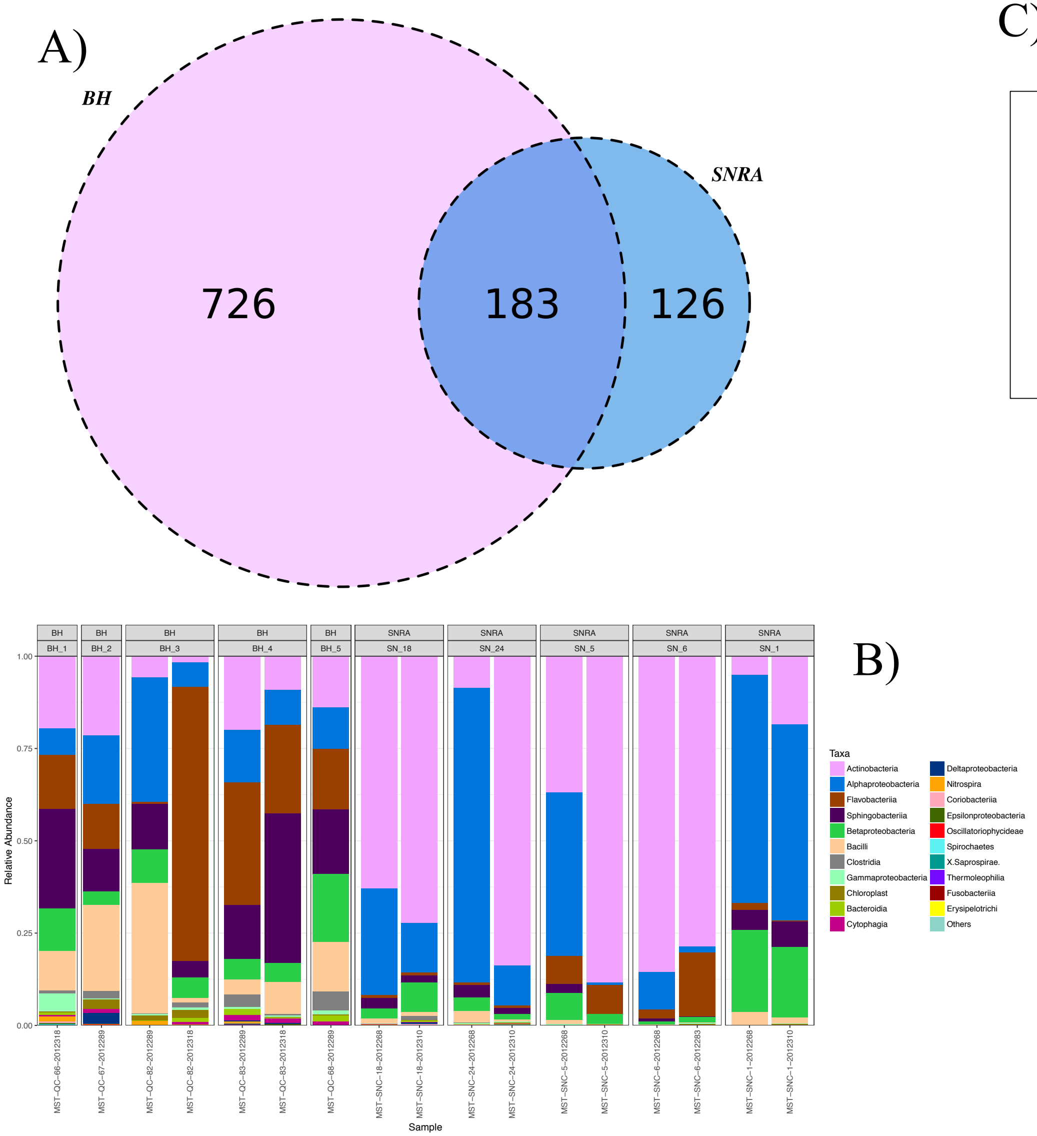

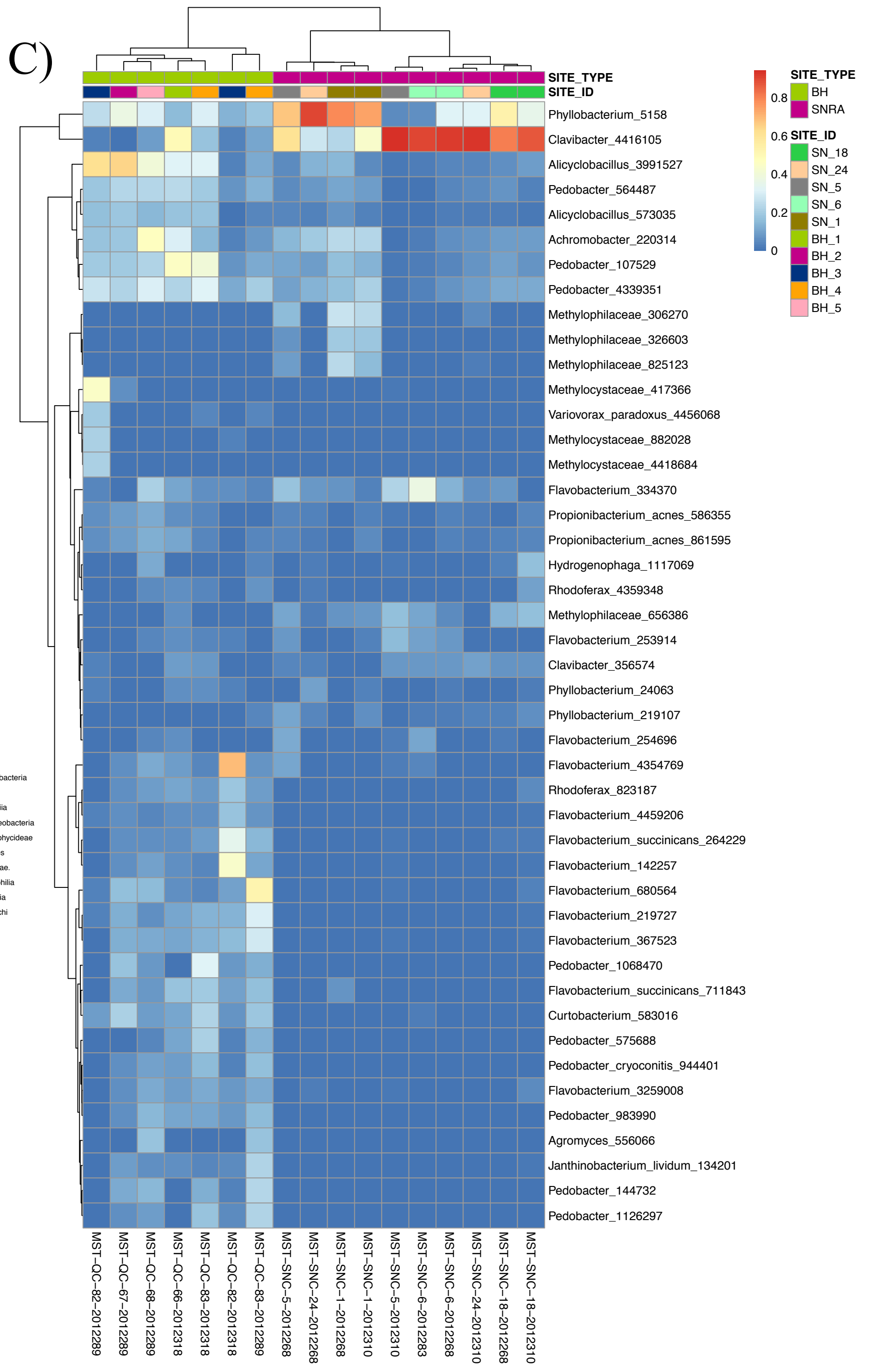

Supplementary Figure S7. Water samples collected at BH and SNRA from fall 2012: A) shared and unique OTUs at BH and SNRA; B) relative abundance of bacterial classes in each sample; C) heatmap of relative abundance of OTUs (> 30 reads) belonging to at least one functional group, using the double square roots of relative abundance matrix.

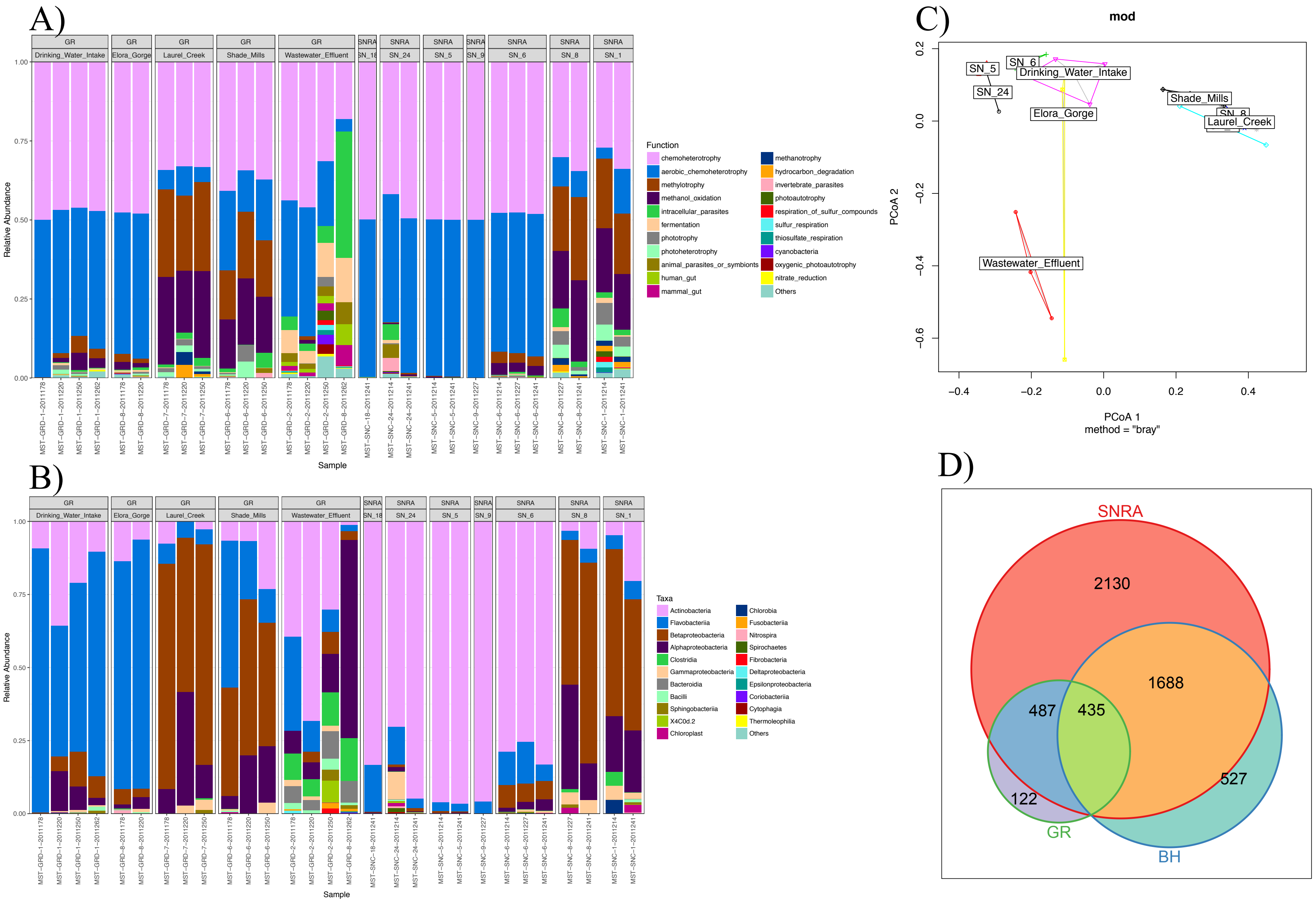

Supplementary Figure S8. A, B&C) Water samples collected at GR and SNRA from summer 2011: A) relative abundance of bacterial functional groups in each sample; B) relative abundance of bacterial functional groups at the class level; C) heterogeneous dispersion of bacterial functional groups using hellinger transformed abundance data. D) Shared and unique OTUs found at BH, SNRA and GR (including all samples).

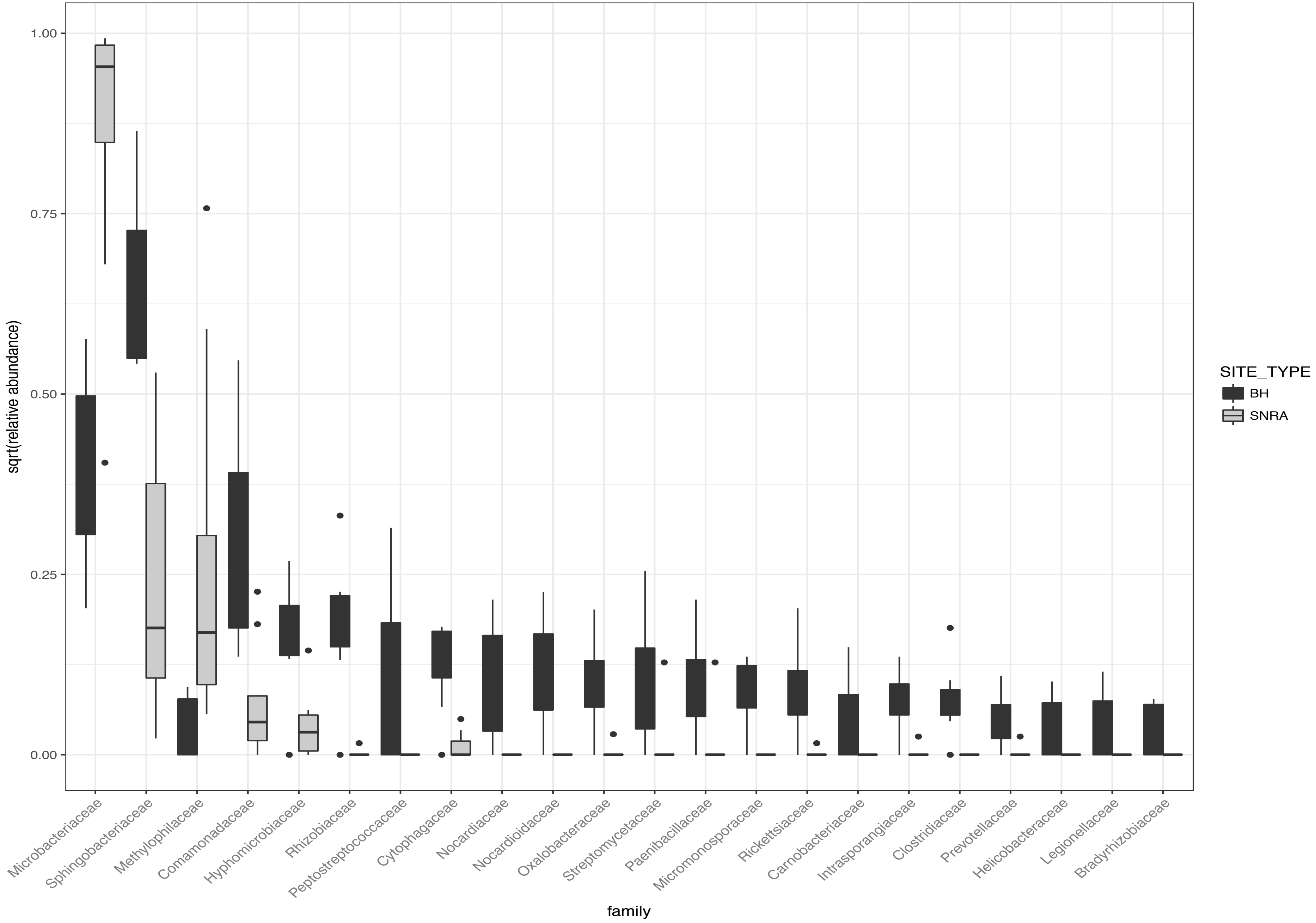

Supplementary Figure S9. Bacterial families differed significantly in relative abundance between the samples collected at BH and SNRA during fall 2012.

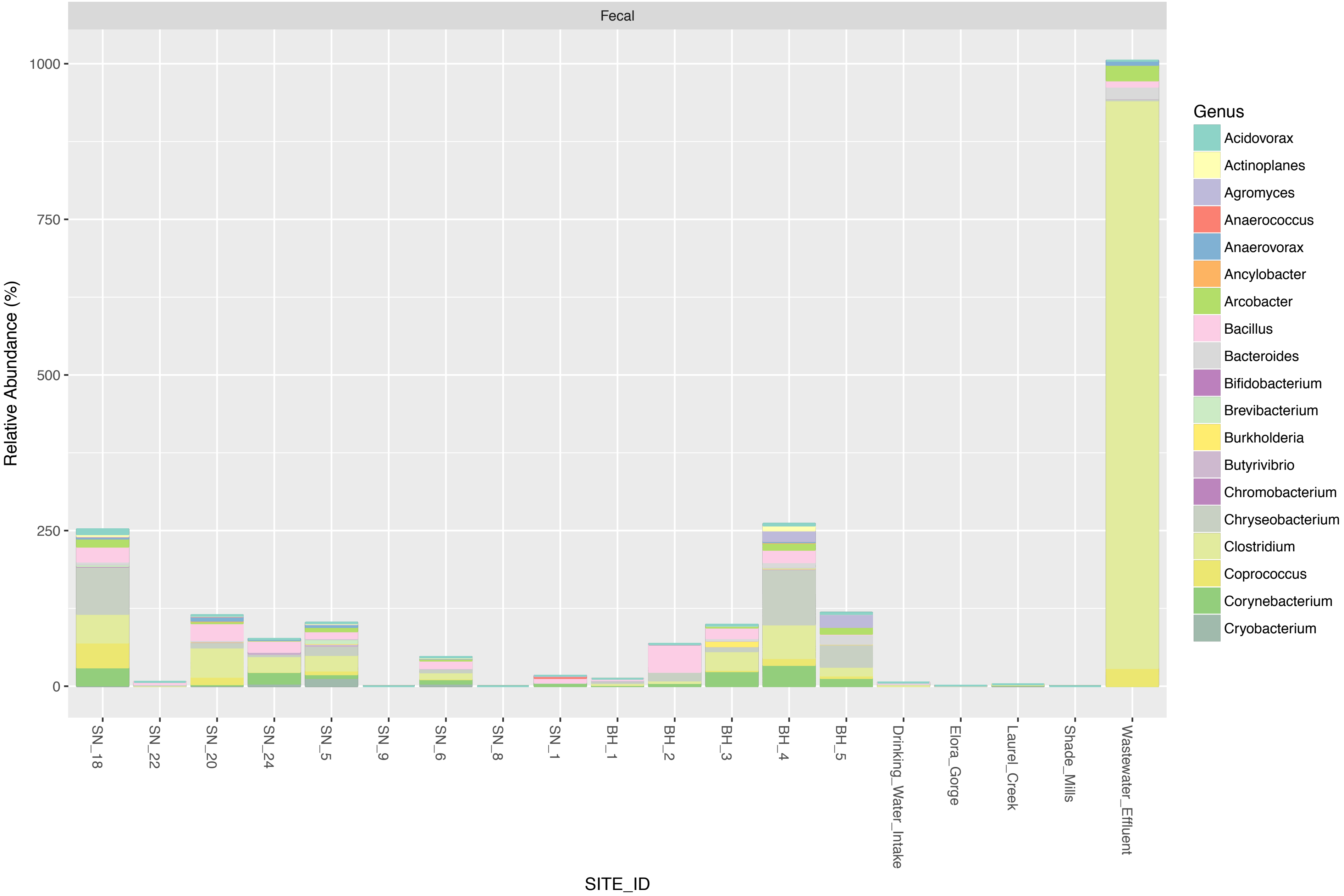

Supplementary Figure S10. Distribution of selected bacterial genera containing fecal indicators.

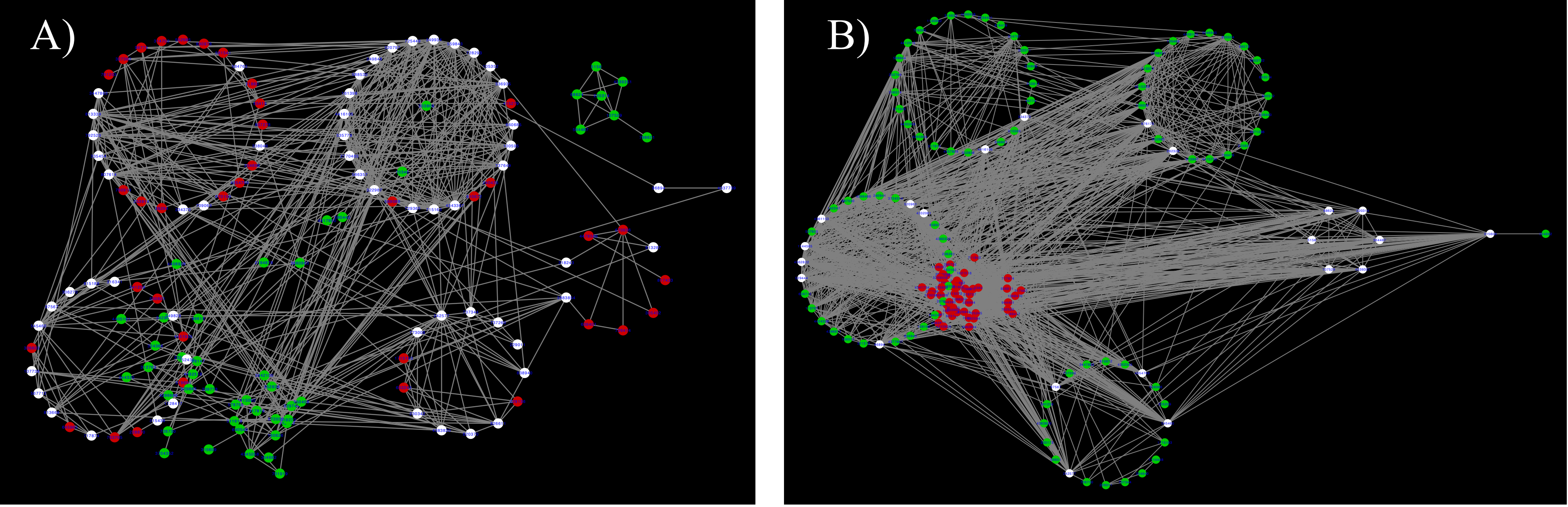

Supplementary Figure S11. Pairwise network comparison: A) shared nodes (white) between the molecular ecological networks (MENs) of GR and SNRA STRAHLER=8&9 (drinking or recreational water) samples; red nodes are only in GR MEN while green nodes are only in SNRA STRAHLER= 8&9 MEN; B) shared nodes (white) between MENs of SNRA STRAHLER = 3&4 and BH (agriculturally dominated watersheds) samples; red nodes are only in BH MEN while green nodes are only in SNRA STRAHLER= 3&4 MEN.

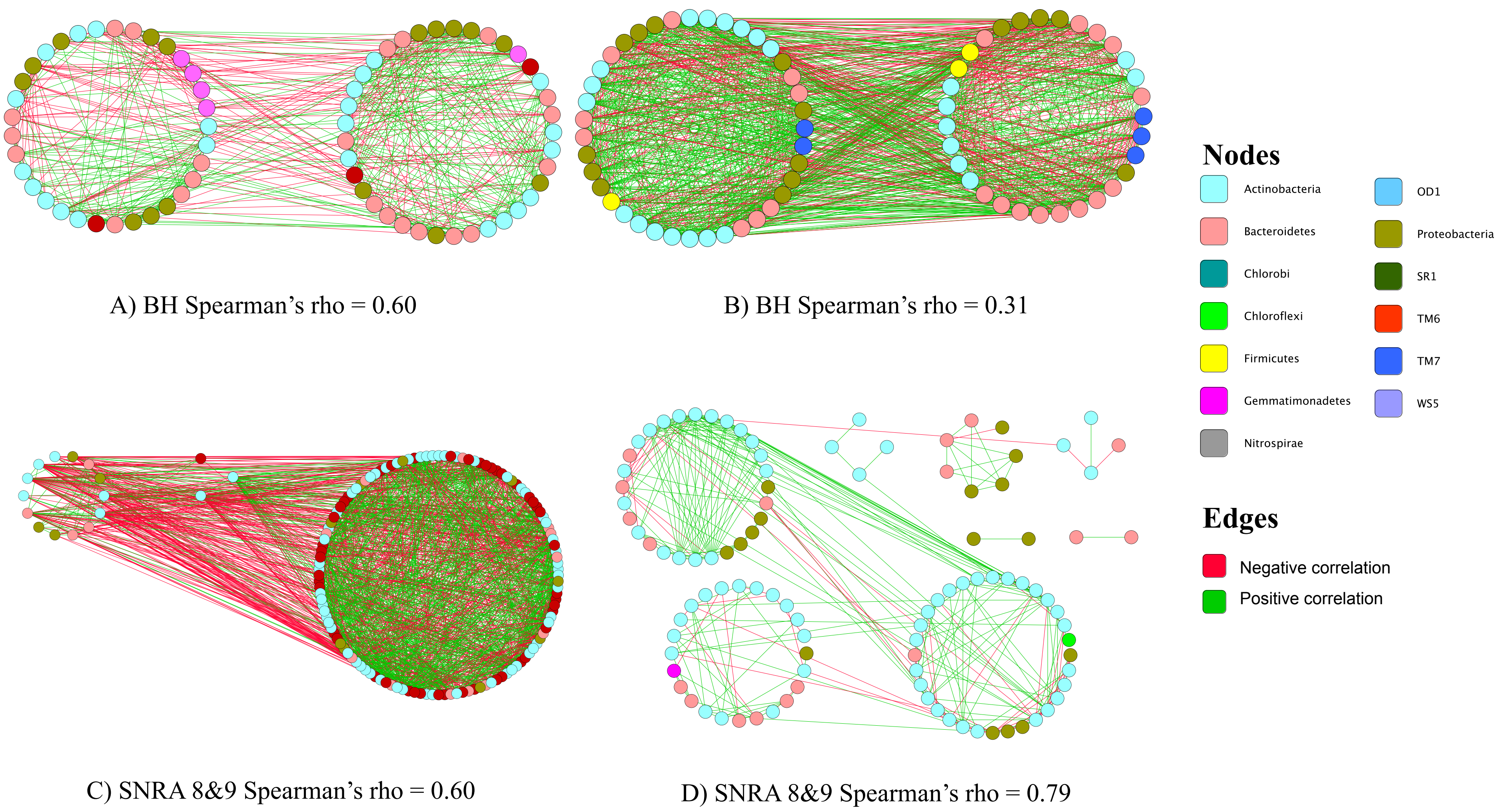

Supplementary Figure S12. Molecular ecological networks (MENs) constructed using arbitrary (A&C, Spearman's rho = 0.60) and RMT-based (B&D, Spearman's rho = 0.31 for B, 0.79 for D) selection of correlation coefficients thresholds. A&B) MENs of BH samples. C&D) MENs of SNRA STRAHLER=8&9 samples. Nodes are colored by phyla; positive and negative correlations are in green and red, respectively.
